# Using landscape history to predict biodiversity patterns in fragmented landscapes

**DOI:** 10.1111/ele.12160

**Published:** 2013-08-11

**Authors:** Robert M Ewers, Raphael K Didham, William D Pearse, Véronique Lefebvre, Isabel M D Rosa, João M B Carreiras, Richard M Lucas, Daniel C Reuman

**Affiliations:** 1 Department of Life Sciences, Imperial College LondonSilwood Park Campus, Buckhurst Road, Ascot, SL5 7PY, UK; 2 School of Animal Biology, University of Western Australia35 Stirling Highway, Crawley, WA, 6009, Australia; 3 CSIRO Ecosystem Sciences, Centre for Environment and Life SciencesUnderwood Ave, Floreat, WA, 6014, Australia; 4 Department of Ecology, Evolution, and Behavior, University of Minnesota100 Ecology Building, 1987 Upper Buford Circle, Saint Paul, Minnesota, 55108, USA; 5 NERC Centre for Ecology and HydrologyWallingford, Oxfordshire, OX10 8BB, UK; 6 Tropical Research Institute (IICT)Travessa do Conde da Ribeira, 9, Lisbon, 1400-142, Portugal; 7 Institute of Geography and Earth Sciences, Aberystwyth UniversityAberystwyth, Ceredigion, SY23 3DB, Wales; 8 Laboratory of Populations, Rockefeller University1230 York Avenue, New York, NY, 10065, USA

**Keywords:** Distance-dissimilarity curve, habitat fragmentation, habitat loss, landscape divergence hypothesis, nested communities, neutral model, random sampling, spatial autocorrelation, spatial insurance, vicariance model

## Abstract

Landscape ecology plays a vital role in understanding the impacts of land-use change on biodiversity, but it is not a predictive discipline, lacking theoretical models that quantitatively predict biodiversity patterns from first principles. Here, we draw heavily on ideas from phylogenetics to fill this gap, basing our approach on the insight that habitat fragments have a shared history. We develop a landscape ‘terrageny’, which represents the historical spatial separation of habitat fragments in the same way that a phylogeny represents evolutionary divergence among species. Combining a random sampling model with a terrageny generates numerical predictions about the expected proportion of species shared between any two fragments, the locations of locally endemic species, and the number of species that have been driven locally extinct. The model predicts that community similarity declines with terragenetic distance, and that local endemics are more likely to be found in terragenetically distinctive fragments than in large fragments. We derive equations to quantify the variance around predictions, and show that ignoring the spatial structure of fragmented landscapes leads to over-estimates of local extinction rates at the landscape scale. We argue that ignoring the shared history of habitat fragments limits our ability to understand biodiversity changes in human-modified landscapes.

## Introduction

The historical pattern of habitat cover has an impact on present-day biodiversity patterns in fragmented landscapes (Harding. 1998; Kuussaari. 2009; Krauss. 2010; Wearn. 2012). This temporal effect occurs because habitat loss and fragmentation may not directly kill individuals of a species, and it can therefore take a number of generations for populations to go extinct after habitat loss. This ‘ghost of land-use past’ (Harding. 1998) can be a powerful force that explains patterns of present-day diversity better than present-day patterns of habitat cover, with the implication that landscape history must now be considered in conservation planning (Schrott. 2005; Dauber. 2006; Kuussaari. 2009).

Such legacy effects can be detected by correlating present-day biodiversity patterns to present and historical patterns of habitat cover (Kuussaari. 2009), with historical impacts inferred when there are significant correlations to previous habitat cover patterns. This approach, however, treats the ‘past’ and the ‘present’ as being distinct and separate categories. It implicitly assumes that habitat change is a process that used to happen but stopped at an undefined point in time, rather than being a cumulative process that operates over many decades and culminates in the present-day landscape. Predicting the magnitude of biodiversity loss arising from this temporal trajectory of land-use change represents a difficult challenge that is exacerbated by failure to consider the cumulative nature of landscape dynamics (Wearn. 2012). The most commonly applied analytical approach to the problem thus far has been the empirical species–area relationship (SAR) for discrete estimates of total habitat loss (Pimm & Askins 1995; Pimm & Raven 2000); the approach can be adjusted to include habitat change as a cumulative rather than a binary process (Wearn. 2012). However, these models still ignore the spatial distribution of species within habitat (He & Hubbell 2011), the geometry of habitat loss in relation to the spatial pattern of species distributions (Pereira. 2012), and the spatial structure of the habitat itself within landscapes. This is despite knowing that habitat fragmentation strongly influences the spatial patterning of biodiversity (Ewers & Didham 2006), and that the accumulation of fine-scale fragmentation effects dictates biodiversity patterns at the landscape scale (Ewers. 2010).

Within a fragmented landscape, the total number of species that persist is a function of how species are distributed among isolated habitat fragments. Any given fragment will likely hold only a subset of all species present in the landscape, and many species will be present in more than one fragment. On this basis, the total number of species that persist in the landscape reflects both the species richness of individual fragments and how many of those species are shared among fragments, with other features of the landscape such as connectivity among fragments also influencing the number of species present in any individual fragment. Species richness within individual fragments in a fragmented landscape can be approximated well by the SAR (Drakare. 2006), but predicting the spatial pattern of shared species among habitat fragments is much more problematic.

An informative neutral model that predicts spatial patterns of biodiversity is an important requirement for inferring the relative importance of non-neutral biological processes (Rosindell. 2011), but despite considerable advances in landscape ecology since the advent of island biogeography theory (Laurance 2008; Fahrig 2003; Didham. 2012), there is still no generalised model that generates neutral predictions of the pattern of shared species in fragmented landscapes. Such neutral predictions are necessary for quantifying the importance of biological processes such as dispersal, and the role of species traits such as body size and trophic level; these are expected to influence the responses of species and communities to habitat loss and fragmentation (Henle. 2004; Ewers & Didham 2006). We demonstrate that explicitly accounting for the history of habitat change within a landscape leads naturally to predictions of shared species among habitat fragments, and these predictions scale up to provide estimates of the number of species expected to be driven extinct from fragmented landscapes given a particular amount and spatial pattern of habitat loss.

Here, we develop a method for quantifying the history of a landscape by treating it as a cumulative rather than a two-step process. We draw heavily on phylogenetic approaches to the evolution of species, treating habitat as a set of ‘lineages’ that have shared ‘ancestry’ that we quantify by recording the historical patterns of connectedness among fragments. This approach generates a ‘terrageny’ of habitat fragments within a landscape that is analogous to a phylogeny of species. Terragenies are developed for two Amazonian landscapes and we adapt phylogenetic metrics to demonstrate the ability to statistically quantify differences in the historical patterns of land-use change between landscapes. We then combine terragenies with a nested random sampling model to generate neutral predictions about biodiversity patterns in fragmented landscapes, including the proportion of species that will go extinct from a given landscape, be shared between any pair of habitat fragments, or be locally endemic to a single fragment. Differences in terragenetic histories are shown to propagate through fragment lineages to generate differences in the expected patterns of biodiversity in the present day. Importantly, we also derive numeric predictions for the variance around each of these predictions and test the terragenetic model using a data set on leaf-litter beetle communities from an Amazonian landscape. We conclude by discussing the set of assumptions that are implicit within the model and the likely impact of relaxing those assumptions on the predictions that arise from the terragenetic model.

## Quantifying Landscape History in a Terrageny

**Figure 1:**
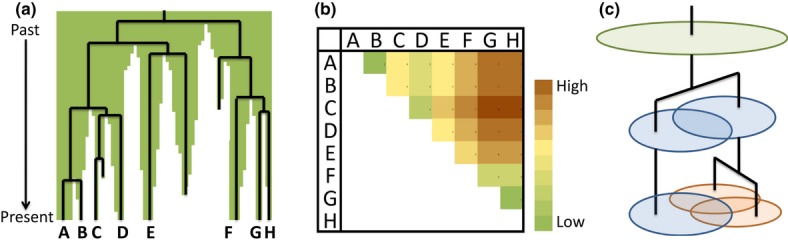
Using landscape history to predict biodiversity patterns. (a) Stylised example of a one-dimensional landscape showing how landscape history can be summarised in a landscape terrageny. Green shading shows habitat cover, which was historically continuous across the landscape. Habitat loss replaces habitat (green) with non-habitat (white), separating the continuous forest area into isolated fragments. This complex landscape dynamic is summarised in a terrageny (black lines). Only habitat fragments that exist in the present-day landscape are labelled (fragments A–H). (b) The terrageny records information on the pattern of shared history among fragments that survived to the present day (fragments A–H), determining their pairwise terragenetic distance. (c) The terragenetic model assumes a single species pool in the original, continuous landscape (green oval). When the forest is divided into two isolated fragments, each fragment retains a random subset of the original species pool (blue ovals) that is progressively sub-divided as fragmentation continues (orange ovals).

We define a terrageny as a record of how a landscape became fragmented through time, containing information on the ‘ancestry’ of fragments and showing how an initially continuous landscape was progressively divided into fragments of decreasing size (Fig.1a). As such, a terrageny has many similarities to a phylogeny of species. At any given point in the history of a landscape, the terrageny provides information on the number and age of extant fragments and their historical spatial relationships. For example, a terrageny provides information on the number of fragmentation events that have separated two fragments, hereafter termed the nodal terragenetic distance between those fragments (Table1; Fig.1b). The nodal terragenetic distance of a pair of fragments, 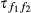, can be quantified most simply as 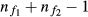, where 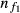 and 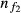 are, respectively, the number of fragment separation events between fragments *f*_1_ and *f*_2_ and their most recent common ancestor fragment. This measure is equivalent to nodal distance in phylogenetics (Gregory 2008; Table1).

**Table 1:** Common metrics of phylogenetic structure and their analogues for quantifying the terragenetic structure of landscapes

Phylogenetic metric	Description	Terragenetic equivalent	Interpretation	Calculation
Mean nodal phylogenetic distance	The mean of the number of nodes separating all pairwise combinations of species on a phylogeny (Gregory 2008)	Median nodal terragenetic distance	The number of nodes quantifies how many fragment separation events occurred in the history of a pair of fragments since they separated from their most recent ancestor fragment. Fragments separated by few fragment separation events are closely related	We calculated a pairwise distance matrix for all fragments on the terrageny, and report the median for terragenies because the distribution was skewed
Mean phylogenetic distance	The mean of the branch lengths separating all pairwise combinations of species on a phylogeny (Webb. 2002)	Median terragenetic distance	Branch lengths give an indication of how recently the two fragments separated from their most recent common ancestor fragment, with long branch lengths indicating fragments that have been separated for a long time period. Thus, fragments separated by large terragenetic distances have been isolated from each other for long time periods	A pairwise distance matrix for all fragments in the terrageny was calculated using the ‘cophenetic.phylo’ function in the R package ‘ape’ (Paradis. 2004). We report the median for terragenies because the distribution was skewed
Topological balance	The extent to which nodes on a phylogeny define subgroups of equal sizes (Mooers & Heard 1997). Balanced phylogenies tend to appear more symmetrical	Terragenetic balance	Terragenetic balance quantifies the degree of asymmetry in a terragenetic tree. A symmetrical tree would suggest that all fragments in a landscape are equally likely to separate into the same number of child fragments, whereas an asymmetrical tree would suggest that some fragments were more likely to separate into child fragments than others	Our terragenies had numerous polytomies, or situations where a fragment splits into > 2 child fragments, so we calculated terragenetic balance with the metric *I*′ (Purvis. 2002), a modified form of Fusco and Cronk's imbalance statistic (Fusco & Cronk 1995). *I*′ was calculated using the ‘fusco.test’ function in the R package ‘caper’ (Orme. 2012)
Evolutionary distinctiveness	The phylogenetic diversity of a clade split equally among its members (Isaac. 2007). Species in older, less-speciose clades are more evolutionarily distinct, while species in younger, diverse clades are less evolutionarily distinct	Terragenetic distinctiveness	High terragenetic distinctiveness indicates fragments that have been isolated from all other fragments for a long time period. It is greatest in fragments that have few siblings or have been separated from other fragments for long time periods	We used the function ‘ed.calc’ in the R package ‘caper’ (Orme. 2012)
Pagel's lambda	The strength of phylogenetic signal in species traits (Pagel 1999). A value of zero suggests trait evolution is independent of phylogeny, a value of one that traits are evolving according to Brownian motion, and intermediate values suggest an effect of phylogeny that is weaker than the Brownian model	Terragenetic Pagel's lambda	We treat fragment size as a ‘trait’ of a fragment, although other physical (e.g. fractal dimension, edge:area ratio) or biological (e.g. species richness, number of local endemics) features could equally be used. If fragments always separated into children that have equal traits, then we would expect closely related fragments to have similar trait values and terragenetic Pagel's lambda to be close to one	We used the function ‘pgls’ in the R package ‘caper’ (Orme. 2012)

To demonstrate how terragenies generate quantitative estimates of landscape history that can provide informative comparisons among landscapes, we constructed terragenies for two landscapes in the Brazilian Amazon and adapted phylogenetic metrics to quantify the terragenetic structure of the two landscapes. Statistical differences in the phylogenetic metrics among the two landscapes are interpreted in light of a detailed understanding of the differences in their land-use history.

### Terragenetic trees in Amazonian landscapes

**Figure 2:**
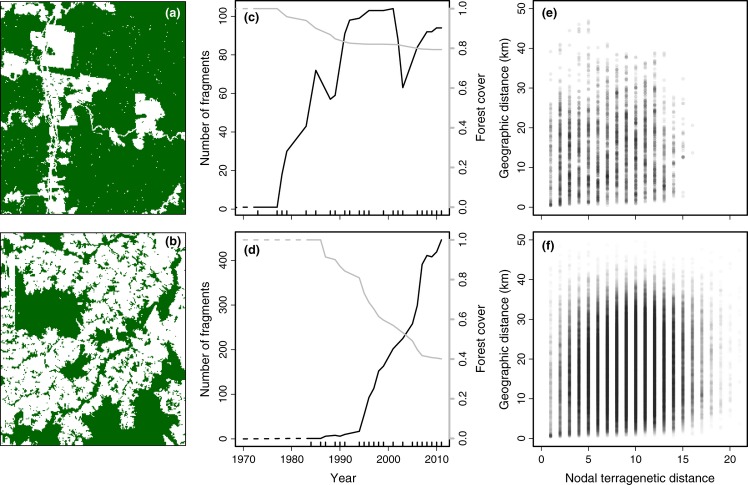
Terragenetic patterns in the Manaus (top row) and Machadinho d'Oeste (bottom row) landscapes. (a,b) Maps of the study landscapes show the present-day (2011) distribution of primary forest (green). Both landscapes are 1254 km^2^ (33 × 38 km). (c,d) Temporal dynamics of the study landscapes through time, as reconstructed from time series maps of land cover. Panels show the number of forest fragments (black line, left axis) and the proportion of forest cover (grey line, right axis) through time. Dashed lines indicate values that were not directly observed. Internal tick marks on the x-axis represent time points when land cover was observed. (e,f) Correlation between nodal terragenetic distance and geographical distance as measured by the distance between fragment centroids. Points are semi-transparent, so darker areas correspond to higher point density.

**Figure 3:**
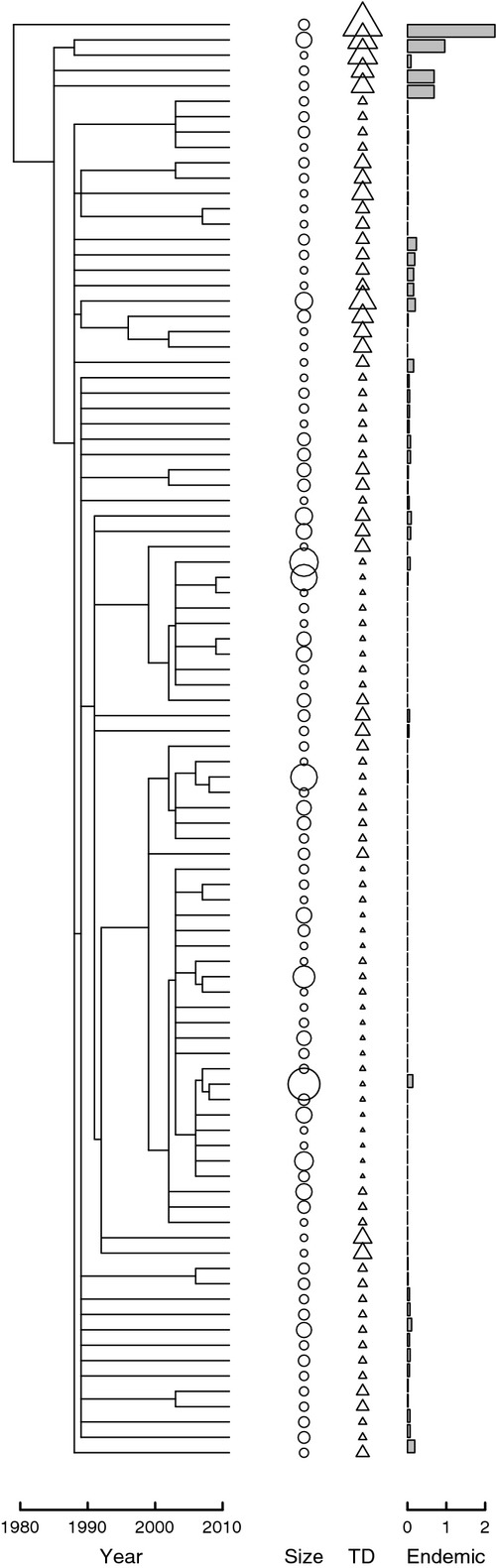
Terrageny for the Manaus landscape. Each horizontal line represents a fragment, with vertical lines connecting sibling fragments to their immediate ancestor. Only the 94 fragments that were present in 2011 are represented. Circles represent log_10_-transformed, present-day size of the forest fragments; triangles represent the terragenetic distinctiveness (TD) of fragments (larger triangles are more terragenetically distinct); the bar chart represents the predicted number of local endemics in each fragment (values were generated using a *z*-value for the SAR of 0.25 and a pool of *s*_0_ = 1000 species). Full terragenies that include all fragments that were destroyed in the Manaus and Machadinho d'Oeste landscapes are presented in Fig. S1.

We constructed terragenies for a 1254 km^2^ landscape in each of the municipalities of Machadinho d'Oeste and Manaus, both in the Brazilian Amazon (Figs2, 3 and S1). The two landscapes were of the same spatial extent (33 × 38 km) and resolution (grid size 150 × 150 m), and, in both, more than 99% of the landscape was covered by forest prior to human encroachment. In both landscapes, we used land cover maps derived from time series of Landsat sensor data following the methods of Prates-Clark. (2009), with maps showing observed forest cover at 23 points in time between 1973 and 2011 in Manaus and at 21 points in time between 1984 and 2011 in Machadinho d'Oeste. While we recognise the role of regrowth in retaining biodiversity, such forests were not included in this analysis and instead we assumed all deforested areas to have remained as such for the duration of the time series.

Deforestation in Manaus began in the early 1970s, accelerated in the mid-1980s and then slowed from the 1990s onwards, resulting in the conversion of 21% of the landscape and the creation of 94 extant forest fragments by 2011 (Fig.2a). Deforestation in Machadinho d'Oeste started in the 1980s, but has progressed more rapidly, leaving a landscape that was 60% deforested with 446 extant forest fragments in 2011 (Fig.2b). The number of habitat fragments in Manaus increased through time in an approximately sigmoidal pattern, although there was a period in the early 2000s when a large number of small fragments were destroyed (Fig.2c). In contrast, forest fragments in Machadinho d'Oeste continue to be created rapidly (Fig.2d).

The median size of fragments in the present day does not differ between the two landscapes (Table2; Wilcoxon test, *W* = 21 992, nominal *P* = 0.442) and neither does the size-distribution of forest fragments (Kolmogorov–Smirnov test, *D* = 0.0743, nominal *P* = 0.784; these are nominal *P*-values because fragment sizes are not independent and, as such, they function as descriptive statistics only, with no probabilistic meaning). Thus, any differences in terragenetic structure between the landscapes will reflect information about landscape structure that is beyond the information contained in present-day distributions of fragment size.

**Table 2:** Summary statistics describing terragenetic structure and biodiversity patterns predicted from the terragenetic model in two landscapes located in the Brazilian Amazon. The tilde (∽) represents statistical models with the response variable to the left and predictor variable to the right. LM represents a linear regression model and PGLS represents a phylogenetic generalised least squares model

Statistic	Landscape	
	Manaus	Machadinho d'Oeste
Fragment size (ha) in 2011	median = 4.5	median = 4.5
	IQR = 2.3–19.7	IQR = 2.3–18.0
Fragment age (years) in 2011	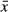 = 11.9	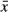 = 5.9
	SD = 7.6	SD = 4.0
Nodal terragenetic distance (τ)	median = 7	median = 9
	IQR = 4–10	IQR = 7–12
Terragenetic distance	median = 44	median = 28
	IQR = 40–46	IQR = 26–30
Pagel's lambda (λ) on log_10_(fragment size) (ha)	λ = 0.0	λ = 0.0
Nodal terragenetic distance (τ) ∽ geographical distance (Mantel test)	*r = 0.08*	*r* = 0.11
	*P* < 0.001	*P* < 0.001
Terragenetic balance (*I*′)	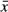 = 0.95	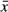 = 0.95
	*P* < 0.001	*P* < 0.001
Terragenetic distinctiveness (TD)	median = 6.6	median = 4.4
	IQR = 5.4–10.8	IQR = 2.7–6.6
Community similarity (Φ) ∽ nodal terragenetic distance (τ) (Mantel test)	*r* = −0.06	*r* = −0.16
	*P* < 0.001	*P* < 0.001
Community similarity (Φ) ∽ geographical distance (Mantel test)	*r* = −0.07	*r* = −0.17
	*P* < 0.001	*P* < 0.001
Local endemics (*u*_*k*_) ∽ fragment creation date (test = PGLS)	F_2,92_ = 27.0	*F*_2,444_ = 37.5
	*P* < 0.001	*P* < 0.001
	*λ = 0.88*	*λ = 0.00*
	*R*^2^ = 0.23	*R*^2^ = 0.08
Local endemics (*u*_*k*_) ∽ fragment creation date (test = LM)	*F*_1,92_ = 16.9	*F*_1,444_ = 37.5
	*P* < 0.001	*P* < 0.001
	*R*^2^ = 0.16	*R*^2^ = 0.08
Local endemics (*u*_*k*_) ∽ fragment separation events (*k*) (test = PGLS)	*F*_2,92_ = 28.6	*F*_2,444_ = 63.8
	*P* < 0.001	*P* < 0.001
	*λ = 0.85*	*λ = 0.00*
	*R*^2^ = 0.20	*R*^2^ = 0.13
Local endemics (*u*_*k*_) ∽ fragment separation events (*k*) (test = LM)	*F*_1,92_ = 16.5	*F*_1,444_ = 63.8
	*P* < 0.001	*P* < 0.001
	*R*^2^ = 0.15	*R*^2^ = 0.13
Local endemics (*u*_*k*_) ∽ terragenetic distinctiveness (TD) (test = PGLS)	*F*_2,92_ = 110	*F*_2,444_ = 114
	*P* < 0.001	*P* < 0.001
	*λ = 1.0*	*λ = 0.0*
	*R*^2^ = 0.54	*R*^2^ = 0.20
Local endemics (*u*_*k*_) ∽ terragenetic distinctiveness (TD) (test = LM)	*F*_1,92_ = 71.1	*F*_1,444_ = 114.0
	*P* < 0.001	*P* < 0.001
	*R*^2^ = 0.44	*R*^2^ = 0.20
Local endemics (*u*_*k*_) ∽ log_10_(fragment size) (ha) (test = PGLS)	*F*_2,92_ = 0.23	*F*_2,444_ = 1.00
	*P* = 0.792	*P* = 0.369
	*λ = 0.59*	*λ = 0.00*
	*R*^2^ < 0.01	*R*^2^ < 0.01
Local endemics (*u*_*k*_) ∽ log_10_(fragment size) (test = LM)	*F*_1,92_ = 0.1	*F*_1,444_ = 1.0
	*P* = 0.762	*P* = 0.318
	*R*^2^ < 0.01	*R*^2^ < 0.01

IQR, interquartile range; SD, standard deviation.

### Applying metrics of phylogenetic structure to terragenetic trees

We adapted and applied standard measures of phylogenetic structure to the two terragenies: terragenetic distance, terragenetic balance, terragenetic Pagel's lambda and terragenetic distinctiveness (Table1; Figs3 and S1). All measures were calculated on terragenetic trees that retained only fragments present in the landscape in 2011.

The frequency distribution of nodal terragenetic distances between fragments (τ) was right-skewed in both landscapes, meaning that most fragments within a landscape have relatively high terragenetic relatedness with a smaller number of more distantly related fragments (Table2). Median terragenetic distance was higher in Manaus than in Machadinho d'Oeste, showing that fragments in Machadinho d'Oeste tend to be more closely related than those in Manaus (Table2). This is likely a consequence of fragmentation having occurred more recently in Machadinho d'Oeste, and hence the median time periods separating habitat fragments were shorter.

Fragments separated by a high nodal terragenetic distance tended to be more widely separated in space (Table2; Fig.2e,f). This occurs because fragment separation creates descendent fragments that are nested within the spatial bounds of the ancestor fragment, so closely related fragments on a terrageny are likely to be, on average, closer geographically than fragments separated by a large terragenetic distance. There is, however, a large amount of scatter around the relationship, showing that terragenies contain information that cannot be inferred from current geography alone.

Both terragenies had significantly unbalanced topologies (Table2), suggesting that when fragment separation events occur, the child fragments tend to have unequal numbers of descendent fragments. Terragenetic Pagel's lambda on the log_10_-transformed fragment sizes had values of λ = 0 in both landscapes, indicating that fragment size is not related to terragenetic history (Table2). Finally, fragments in Manaus had significantly higher median terragenetic distinctiveness than in Machadinho d'Oeste (Table2; Wilcoxon test, *W* = 31 934, nominal *P* < 0.001), reflecting the longer history of deforestation in the landscape and the fact that individual fragments tend to be older in Manaus (Wilcoxon test, *W* = 30 909, nominal *P* < 0.001; Table2).

## From Landscape History to Spatial Patterns of Biodiversity

We here develop a neutral model that uses the history of a fragmented landscape, summarised in a terrageny, to predict biodiversity patterns and the variance around those patterns. Predictions are generated for the two Amazonian landscape terragenies presented above, allowing us to demonstrate how differences in historical patterns of habitat loss and fragmentation accumulate through fragment lineages to generate predicted differences in the spatial patterns of biodiversity in the present-day landscapes. We use the term ‘neutral’ to highlight the fact that we make an assumption of ecological equivalence, as done in other models which have collectively come to be known as ‘neutral’ models (sensu Gotelli & McGill 2006). In our model, neutrality is at the level of species, as it is in the Theory of Island Biogeography (MacArthur & Wilson 1967; Gotelli & McGill 2006), rather than at the level of individuals as in the Unified Neutral Theory of Biodiversity and Biogeography (Hubbell 2001).

Our goal is to overlay a random sampling model onto a terrageny to build a neutral expectation for the proportions of species that will go extinct from a given landscape, be shared between any pair of habitat fragments, or be locally endemic to a single fragment (Fig.1c). To predict biodiversity patterns, we use a single biological parameter, a *z*-value for the SAR of 0.25, which is commonly employed to predict species responses to habitat loss and fragmentation (Pimm & Askins 1995; Pimm & Raven 2000; Venter. 2009; Wearn. 2012). Formal derivations of all equations are presented in the Online Appendices (Supporting Information).

The model begins with a single species pool that is present in a continuous landscape (Fig. 1c). As habitat loss and fragmentation progress, the continuous landscape is divided into isolated ‘child’ fragments and we use the SAR to predict the proportion of the species pool that will persist in each fragment. The total number of species that persist in the fragmented landscape is given by the sum of species richness in each fragment, minus the species that are shared among fragments. Because habitat has been lost, the total number of species persisting in the fragments is lower than it was in the original species pool, meaning local extinctions have occurred. We assume species are randomly distributed among child fragments, and that a ‘grandchild’ fragment can only inherit species from its parent. Through repeated fragment separation events that more finely divide the habitat within the landscape, this repeated random sampling from parent fragments can lead to species being confined to a single fragment within the landscape, becoming endemic to that particular fragment.

### Variance around extinction estimates following habitat loss

The SAR predicts that the number of species a given habitat fragment can support is determined by its size (Rosenzweig 1995), typically via a relationship of the form 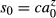 where *s*_0_ is number of species, *a*_0_ is fragment size, and *c* and *z* are constants (Drakare. 2006). Following habitat loss, habitat area is reduced to the proportion *a*/*a*_0_ of the original area *a*_0_, and the proportion *s*/*s*_0_ = (*a*/*a*_0_)^*z*^ of the species originally present in the landscape persist (Pimm & Askins 1995). Under this model, the proportion of species retained in the modified landscape is fixed and there is zero variance, reflecting the assumption that the species ‘capacity’ of the reduced amount of habitat is fixed and therefore that the probability of a particular species being retained is not independent of the probability of all other species (i.e. if one species persists, then it increases the probability that another species will not persist).

**Figure 4:**
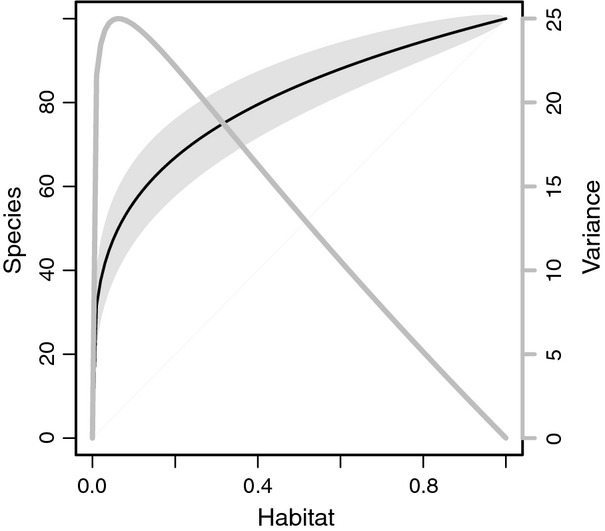
Predicted reductions in species richness following habitat loss according to the species–area relationship. Habitat and species richness are represented as proportions. The black line (left axis) represents the mean number of species expected to persist in relation to the proportion of habitat that is retained in the landscape, and light grey shading shows the area encompassed by the 95% confidence interval around the prediction. The grey line (right axis) illustrates variance around the species richness estimates. Values were generated using a *z*-value for the SAR of 0.25 and a pool of *s*_0_ = 100 species.

In a fully neutral model, we might assume that each species has an independent probability of persisting in the modified landscape (Didham. 2012), with that probability given by re-interpreting the *z*-value of the SAR as the accumulation of species-level events rather than a community-level constant. Under this interpretation, the probability of any given species surviving following habitat loss is given by *p* = (*a*/*a*_0_)^*z*^. Each species in the original landscape now represents an independent Bernoulli trial, *F*_*i*_, with a probability of success *p* and a probability of failure *q* = 1 − *p*, and species richness is measured in positive integers rather than as a proportion of the original species pool. This follows a binomial distribution, with the number of species persisting following habitat loss, *S*_*l*_, given by the sum 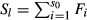. This has expected value *s*_0_*p* and variance *s*_0_*p*(1 − *p*). Substituting *p* = (*a*/*a*_0_)^*z*^, we obtain the original expectation that *s*_0_(*a*/*a*_0_)^*z*^ species will persist following habitat loss, but with the benefit of having now obtained the variance around that expectation as 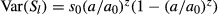 (Appendix A3). When *z* = 0.25, variance is highest when *c*. 95% of the original habitat has been lost (Fig.4).

### Predicting extinction in fragmented landscapes

For a fragment *f*_*k*_, there is a proportion of species that were in *f*_*k*_ but are not in any present-day descendant fragments of *f*_*k*_: they go extinct from the fragment lineage of *f*_*k*_ before the present day. If fragment *f*_*k*_ splits into child fragments 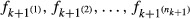 then the expected proportions *e*_*k*_ (respectively, 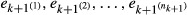) of species in *f*_*k*_ (respectively, in 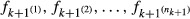) that go extinct by the present day follow the recursive formula

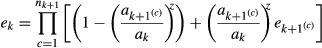
where 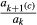 is the proportion of the area of *f*_*k*_ that remains in 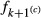 (Appendix A7). The sum in the brackets is the expected proportion of species in *f*_*k*_ that do not survive to occupy 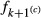, plus those that do, but subsequently go extinct from the lineage of 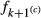.These sums must be multiplied because extinction from the lineage of *f*_*k*_ requires the species to either not be present in, or go extinct from, each of the fragment lineages descending from *f*_*k*_. The recursive base case, in which fragment 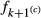 does not split into any child fragments, corresponds to 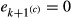. The expected proportion of species that go extinct from the full landscape is obtained by setting *f*_*k*_ to the fragment that represents the entire landscape, *f*_0_, at the root of the terrageny. The variance around *e*_0_, the expected proportion of species in the original landscape that go extinct, can be quantified by considering extinction as a sum of Bernoulli trials across the *s*_0_ species that are present in *f*_0_, and equals 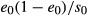 (Appendix A7).

Predicted local extinctions in the Manaus and Machadinho d'Oeste landscapes were very low, because the pattern of shared species among non-randomly distributed habitat fragments generates a form of spatial insurance (Loreau. 2003). For a taxon with *s*_0_ = 1000 species in the original landscapes, the model predicted that habitat loss and fragmentation would have caused the local extinction of just 3.2% (95% confidence interval 2.2–4.4%) of the original species pool from the Manaus landscape and 5.1% (95% CI 3.8–6.5%) in the Machadinho d'Oeste landscape. These values are lower than the 5.7% (95% CI 4.3–7.2%) and 20.5% (95% CI 18.0–23.0%) loss predicted from applying the SAR to the total amount of habitat in the two landscapes, respectively, and highlight the importance of considering the fate of biodiversity within individual fragments (Ewers. 2010). The confidence intervals of the terragenetic and landscape SAR predictions overlap in Manaus but not in Machadinho d'Oeste, suggesting that the importance of modelling landscape history becomes more pronounced in more fragmented landscapes.

### Predicting shared species among habitat fragments

If *f*_*k*_ and *f*_*h*_ are two fragments with most recent common ancestor fragment *f*_*r*_, and if *a*_*k*_, *a*_*h*_, and *a*_*r*_ are the areas of these fragments, then the fragments *f*_*k*_ and *f*_*h*_ are expected to share a proportion 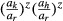 of the species that were present in *f*_*r*_ (Appendix A4). The proportion

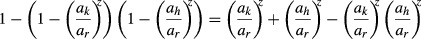
of the species in *f*_*r*_ will be retained in one or the other of the fragments (Appendix A5), and hence the expected proportion of the species that are shared between the two fragments is

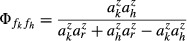
(Appendix A6). This is directly equivalent to Jaccard similarity, a commonly used measure of overlap in community composition (Magurran 2003; Chao. 2005). Variance around this expectation is also available online (Appendix A6).

**Figure 5:**
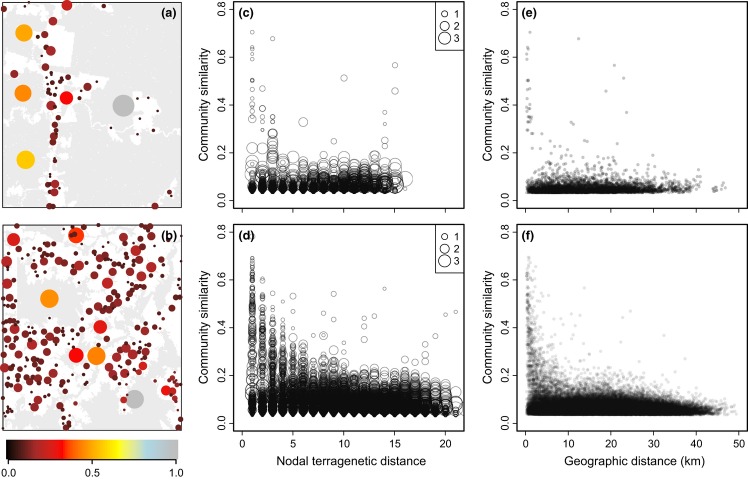
Predicted biodiversity patterns arising from the terragenetic model in the Manaus (top row) and Machadinho d'Oeste (bottom row) landscapes. Predictions were made using a *z*-value for the species–area relationship of 0.25. (a,b) Spatial pattern in community similarity among habitat fragments. Colours represent the proportion of species shared with the largest fragment in each landscape (grey circle), and are plotted at the centroid of each fragment. The size of points reflects log_10_-transformed fragment size. (c,d) Predicted community similarity against nodal terragenetic distance, with point size reflecting the log_10_ ratio of fragment sizes. (e,f) Predicted community similarity against geographical distance. Geographical distance is measured as the distance between fragment centroids and points are semi-transparent, so darker areas correspond to a higher density of points. In panels c–f, community similarity is represented as Jaccard similarity and represents the proportion of species that are common to any given pair of habitat fragments.

We compared the community in each fragment with the community observed in the largest fragment within the respective landscapes and mapped community similarity across the two landscapes in the Brazilian Amazon (Fig.5a,b). Predicted community similarity declined with terragenetic distance in both landscapes (Table2), although there was a large amount of scatter around that relationship (Fig.5c,d). One of the limitations of Jaccard similarity is that a small fragment with few species can only share few species with a large fragment that has many species, thus the size difference between a pair of fragments sets an upper limit to the community similarity of that pair. We illustrated this effect by plotting the log_10_ ratio of fragment sizes [log_10_(area of largest fragment/area of smallest fragment)], finding that fragment pairs with the highest community similarity were those that were more similar in size (Fig.5c,d). Together, these results indicate that both the terragenetic history and the present-day distribution of fragment sizes play important roles in determining the present-day pattern of shared species among habitat fragments.

### Spatial autocorrelation and community turnover

As the communities of descendent fragments are nested subsets of the community in the ancestor fragment, the terragenetic model predicts that closely related fragments will have more species in common than fragments widely separated on the terrageny (Fig.5c,d). Moreover, closely related fragments also tend to be close in geographical space (Fig.2e,f), so the terragenetic model should also predict that fragments located close together in space are likely to have more species in common than fragments widely separated in space. This negative correlation was statistically significant in both landscapes (Table2), with the terragenetic model predicting a very rapid decline in community similarity over small spatial scales followed by low similarities over larger distances (Fig.5e,f). Combined with the nested structure of communities in descendent fragments, this pattern may help explain the landscape divergence phenomenon (Laurance. 2007), in which communities inhabiting fragments within the same landscape (i.e. close together) appear to have convergent species compositions whereas those in different landscapes tend to be divergent.

### The location of locally endemic species

We define a locally endemic species as a species that is present in just one habitat fragment within the landscape. Information on the likely locations of local endemics is of immediate conservation interest at local scales in much the same way as the locations of globally endemic species are important for identifying areas of conservation priority at global scales (Wilson. 2006). The terragenetic model predicts the expected proportion, *u*_*k*_, of species from the original, full landscape, *f*_0_, that end up locally endemic to a present-day fragment, *f*_*k*_, that is separated from *f*_0_ by *k* fragmentation events. If event *t* (*t* = 1,…,*k*) consists of a single separation into fragment 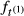, the direct ancestor of *f*_*k*_, and the siblings of 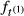, here denoted 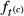 for *c* = 2,3,…,*n*_*t*_, then

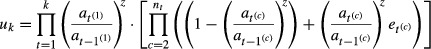
where 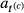 is the area of 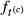 (Appendix A8). The left product is over all separation events leading to *f*_*k*_ because in order for a species to be endemic in *f*_*k*_ it must have survived, on each fragmentation event, *t*, leading to *f*_*k*_, to occupy 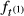 (probability given by the term 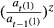). The species must also have failed to survive to the present day in the lineages descending from the sibling fragments 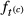 (probability given by the product in the bracket). The product in the bracket resembles the extinction equation of an earlier section and follows a similar logic. Variance around the expectation given above is also available online (Appendix A8).

Estimates of *u*_*k*_ are always greater than zero, but median values were very low (< 1 × 10^−4^) for our two landscapes (Figs3 and S1), suggesting few fragments in either landscape are likely to have locally endemic species. The highest predicted values were 0.002 in a single fragment in Manaus, and 0.004 and 0.001 in two fragments, respectively, in Machadinho d'Oeste. These values suggest that for diverse taxa such as invertebrates with *s*_0_ = 1000 species within the landscape, there is one fragment in Manaus that is expected to contain two locally endemic species, while in Machadinho d'Oeste one fragment is expected to contain four local endemics and another is expected to contain one. Other fragments with lower values of *u*_*k*_ may also contain local endemics, each with low probability.

We used phylogenetic generalised least squares to account for the non-independence of related fragments to examine the correlates of predicted local endemic species richness. Phylogenetic generalised least squares models were calculated using the function ‘pgls’ in the R package ‘caper’ (Orme. 2012). We found that the number of predicted local endemics was higher in fragments that arose earlier in the terrageny and in fragments that had fewer fragment separation events in their history (Table2). As expected, we found that more terragenetically distinct fragments were more likely to contain more local endemics in both landscapes (Table2). In contrast, the proportion of local endemics was not significantly related to log_10_-transformed fragment size (Table2), suggesting that the presence of local endemics is related more to the temporal dynamics of historical patterns of habitat change than it is to the present-day structure of habitat in the landscape.

Interestingly, the phylogenetic analyses in the Manaus landscape had λ values approaching one, whereas the λ values in Machadinho d'Oeste were zero (Table2). Values approaching λ = 1 indicate that model residuals show significant terragenetic structuring, suggesting that in the Manaus landscape standard statistical methods such as linear regression, which treats fragments as independent replicates for analysis, would incorrectly estimate the model parameters. Indeed, linear regression models testing the same relationships above always had lower explanatory power than phylogenetic generalised least squares models in the Manaus landscape, but there was no difference among linear and phylogenetic models fitted in the Machadinho d'Oeste landscape (Table2).

### Model validation

We tested the ability of the terragenetic model to predict biodiversity patterns using data collected on leaf-litter beetle (Coleoptera) communities at the Biological Dynamics of Forest Fragments Project (Didham. 1998a) located within the Manaus landscape modelled above. Although the beetle data are not an ideal complete census of many fragments within the landscape, the full data set did encompass 8494 individuals from 993 species collected in seven fragments ranging in area from one hectare through to continuous forest. Using these data, we estimated the *z*-value of the SAR to be 0.11, which was the value employed to make all terragenetic predictions in this case. Beetle communities were sampled in 1994, and thus we trimmed our terrageny to make terragenetic predictions for the landscape as it was in the year 1994. Data on beetle communities were available for just four of the 1994 fragments represented in the historical land-use terrageny for Manaus (out of a total of seven fragments in the original beetle data set – the remaining three were so small they were below the mapping resolution used to construct the terragenies), giving a combined sample size of 7976 beetles from 947 species. Even with these large sample sizes, the observed number of species per fragment was less than 60% of the number of species predicted to be present by the Chao1 diversity index (range 24–60%), and this undersampling influences the calculation of similarity indices and the estimation of numbers of locally endemic species in this data set (Didham. 1998b). To mitigate this, we used rarefaction to generate estimates of community composition for a standardised sample size. We randomly assigned 500 individuals to species within each fragment, with assignations weighted by the observed relative abundance of the species within that fragment. We repeated this process 1000 times, and we tested the terragenetic predictions for each of the 1000 rarefied site × species matrices using Pearson correlations. This process allowed us to generate mean and 95% confidence intervals around the correlation coefficients used to test the terragenetic predictions.

**Figure 6:**
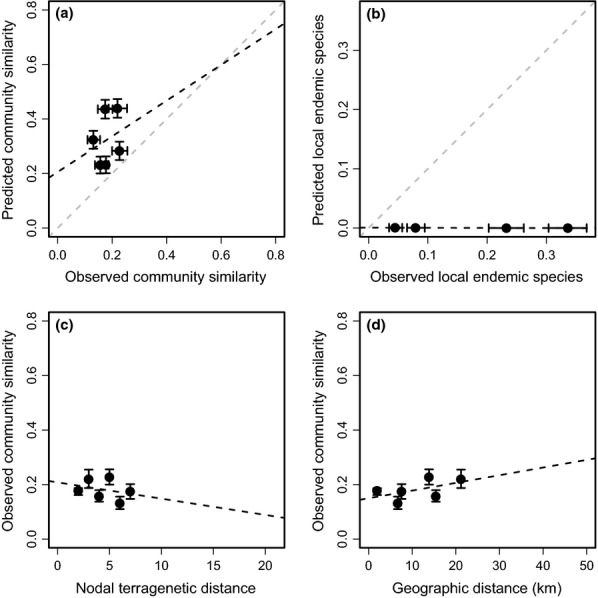
Empirical validations of the ability of the terragenetic model to predict patterns of leaf-litter beetle community composition in the Manaus landscape. (a) Observed vs. predicted community similarity and (b) observed vs. predicted proportion of locally endemic species. Grey dashed line shows the 1 : 1 relationship that would be followed if the model made perfect predictions. Observed community similarity (c) declines with increasing terragenetic distance between fragments but (d) increases with geographical distance between fragments. In all panels, black dashed lines show the relationship fitted using linear regression. Error bars represent the 95% confidence interval around predicted and observed values. Terragenetic predictions were generated using a *z*-value for the SAR of 0.11. Community similarity is represented as Jaccard similarity and represents the proportion of species that are common to any given pair of habitat fragments.

As expected under the terragenetic model, we found a positive correlation between predicted and observed community similarity (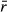 = 0.25, 95% CI = −0.07 to 0.54; Fig.6a) and a strong, negative correlation between observed community similarity and terragenetic distance (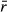 = −0.28, 95% CI = −0.51 to −0.01; Fig.6c). Contrary to the terragenetic predictions, we found a positive correlation between community similarity and geographical space (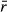 = 0.52, 95% CI = 0.28 to 0.72; Fig.6d), although the slope was near-zero (slope = 0.003) and, with just four fragments, we were unable to reject the hypothesis that the observed slope was zero (linear regression: *F*_1,4_ = 1.66, *P* = 0.27). Furthermore, this pattern may have been an artefact of a weak negative correlation between geographical distance and the log-ratio of fragment sizes for the four fragments in this analysis (Mantel test: *r* = −0.08, *P* = 0.58). We also detected a negative correlation between observed and predicted local endemic species richness (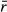 = −0.92, 95% CI = −0.96 to −0.87; Fig.6b). However, the slope of this relationship was also near-zero (slope = −0.002) and observed numbers of endemic species were several orders of magnitude higher than the predictions, likely an artefact of sampling just four fragments incompletely. Many species deemed locally endemic in our empirical data will be shared with fragments that were not sampled, and this undersampling of fragments will greatly inflate the observed number of local endemics. Undersampling of the beetle communities within the fragments probably also contributes to the inflated estimates of endemic species. This interpretation is supported by the low values of observed community similarity in the beetle data (Didham. 1998b), with observations being consistently lower than the values predicted under the terragenetic model.

## Discussion

The terragenetic model is a tree-based model that is neutral at the level of species and allows us to predict the spatial patterns of biodiversity in human-modified landscapes, providing a mathematical representation of how historical habitat change can leave a spatial signature on present-day biodiversity. It is more informative as a null model than MacArthur & Wilson's (1967) Theory of Island Biogeography because it predicts spatially explicit patterns of shared species among fragments rather than species richness within fragments alone. Unlike the Unified Neutral Theory of Biodiversity and Biogeography (Hubbell 2001), it does not predict the species abundance distribution, and neutrality in the terragenetic model is at the level of species rather than individuals.

A terrageny represents a quantitative measure of landscape history. It combines the amount of habitat loss, the patterns of habitat fragmentation and the historical changes in both of these features into a single, unique description of landscape structure. We believe this is particularly important given the interrelated nature of habitat loss and habitat fragmentation (Fahrig 2003; Didham. 2012). The approach of representing landscape history as a terrageny integrates these dual processes and patterns, and provides a natural position from which to make predictions about their joint effects on biodiversity. Importantly, the terragenies quantified differences between the landscapes despite landscape statistics such as the size-distribution of habitat fragments suggesting there was no difference, and provided information about individual fragments that was additional to that obtained from examining physical features such as fragment size and geographical proximity to neighbouring fragments. These differences propagate through the terragenetic model to change our expectation of extinction rates in fragmented landscapes, and generate predictions about the possible locations of locally endemic species.

### Using the species–area relationship to predict extinction rates

By applying SAR predictions at the scale of individual fragments rather than the landscape as a whole, the terragenetic model retains the basic philosophy of SAR-based approaches to estimating extinction rates. The fundamental difference in approach mirrors the difference between continental and island-based SARs (Rosenzweig 1995; Drakare. 2006): landscape-based SARs rely on a continental SAR and effectively combine all remnant habitat into a single estimate of habitat amount, whereas the terragenetic model is built from an island SAR that estimates the number of species retained in individual habitat fragments. The terragenetic model thereby retains biologically relevant information on the size-distribution of fragments within landscapes as it makes its predictions (Ewers. 2010).

The continental SAR has been criticised for over-estimating extinction rates that may be better approximated by an endemics–area relationship (EAR) (He & Hubbell 2011). However, whether the SAR or EAR is most appropriate may depend on the geometry of habitat loss and how that intersects the spatial distribution of species (Pereira. 2012). Both of these approaches make the unrealistic assumption that all remnant habitat is spatially discrete, whereas the terragenetic model allows for fragmented landscapes to have complex spatial patterns consisting of multiple habitat remnants. Importantly, the terragenetic approach also provides much more information than a landscape-scale application of the continental SAR or EAR. The continental SAR and EAR predict the total number of species that should be retained in a landscape assuming all remnant habitat is continuous, and cannot be down-scaled to predict the distribution of species within a landscape. In contrast, the terragenetic model uses the island SAR to predict the spatial distribution of species among the various fragments that comprise the habitat retained within the landscape, and can be up-scaled to predict the total number of species persisting within a landscape. Moreover, we obtain these additional predictions without having to add additional biological parameters to the model.

We also re-interpreted the SAR as providing a species-level probability rather than a community-level quantity, thereby allowing us to calculate the variance around all of our predictions of biodiversity patterns (Online Appendices). SAR-based estimates of extinction are sometimes accompanied with variance estimates, but only by assuming there is variance in the *z-*value itself (e.g. Venter. 2009; Wearn. 2012). In contrast, we assumed that *z* reflects the community-level outcome of a stochastic process that operates at the level of individual species. This allowed us to predict the level of variance around SAR-based extinction estimates, a new approach that may help provide resolution on debates about the utility of large-scale estimates of extinction made using the SAR (Pimm & Askins 1995; Pimm & Raven 2000; He & Hubbell 2011; Wearn. 2012).

### Assumptions of the terragenetic model

We constructed the terragenetic model using a set of simplifying assumptions, of which we believe four are particularly important. First, we assumed that all species were distributed randomly across the pre-fragmentation landscape. However, pre-existing environmental heterogeneity and species turnover make this unlikely and are known to influence the outcomes of SAR-based predictions of extinction (He & Hubbell 2011; Pereira. 2012). This will likely lead to communities sharing less species than predicted by the terragenetic model. Second, the model has assumed that fragment size is a constant until such a time as it separates into two sibling fragments. But we know that accounting for cumulative habitat loss that gradually erodes the size of an individual fragment can impact predicted extinction rates (Wearn. 2012). A related issue is that we assumed species richness reaches equilibrium instantaneously when in fact this process can take many decades and can be dependent on the size of the fragment (Brooks. 1999; Ferraz. 2003; Halley & Iwasa 2011). If a fragment separates before equilibrium species richness is reached, it will pass on more species to its descendent fragments than accounted for in our models, and by ignoring this extinction debt the model may underestimate the number of species that remain in landscapes undergoing rapid change. Third, dispersal among fragments is a key component of biodiversity persistence in fragmented landscapes (Hanski 1998) and results in species occupancy patterns shifting among fragments, but was not incorporated in the model. Over long time periods, continuous dispersal may erase any terragenetic signature from patterns of shared species. Finally, the terragenetic model assumed neutrality at the level of species, so non-random patterns of species susceptibility to habitat loss and fragmentation might generate nested communities (Henle. 2004; Ewers & Didham 2006; Watling & Donnelly 2006) that are more similar than predicted by the terragenetic model. Similarly, we made the simplifying assumption that all species were habitat specialists and did not persist in, or disperse through, the matrix, despite considerable evidence to the contrary for some groups (Kupfer. 2006).

### Testing the terragenetic model

We used pre-existing data on leaf-litter beetle communities to validate the terragenetic model, relying on one of the largest existing data sets on the responses of invertebrate communities to habitat fragmentation collected in the tropics (Didham. 1998a). Even this data set, however, had relatively little power to test the terragenetic model for two reasons. First, beetles were sampled at 44 different locations in the Manaus landscape, but that corresponded to just four separate fragments for which we also had terragenetic data. This arose because samples were collected along edge gradients within fragments and because the four control sites used in the original study were all located within different regions of the same continuous area of forest. Second, the difficulties of fully censusing such diverse communities ensured the communities in all fragments were undersampled (Didham. 1998b), reducing the certainty with which patterns of relative community composition can be quantified. Notwithstanding these issues, it was encouraging to note that the observed spatial patterns of among-fragment community similarity and similarity declines with terragenetic distance were broadly consistent with the predictions of the terragenetic model.

Testing the predictions of the terragenetic model with higher resolution empirical data represents a challenging, but achievable, exercise for future work. The basic unit of biodiversity information that is predicted by the model is community similarity in the form of Jaccard similarity. This measure lends itself to field validation, although it can be difficult to rigorously quantify as it would ideally require a complete census of the community inhabiting each fragment, as demonstrated by our preliminary validation attempt. Jaccard similarity is also limited by the size-difference of the fragments being compared, but remains the most parsimonious metric for use in the terragenetic model. The terragenetic model does not predict the abundance of species so abundance-based similarity indices cannot be used, and Jaccard similarity is more easily interpreted than the widely used Sørensen similarity index that is monotonically related to Jaccard (Chao. 2005). Field-based studies of habitat fragmentation typically subsample communities rather than census them (Nufio. 2009; Stork. 2009), although methods do exist to use abundance data to account for undersampling when communities have not been fully censused (Chao. 2005). Even so, it will be necessary to heavily sample a large number of fragments across an entire landscape to gain enough among-fragment comparisons of shared species to provide a meaningful test of the model. The size of the landscape itself should also be carefully defined to ensure that terragenetic predictions are being made at spatial scales that are appropriate to the particular taxon being studied. The sampled fragments in field studies are themselves chosen to examine gradients of features such as landscape habitat amount and fragment size, but to test the terragenetic model it will be necessary to structure the collection of field data in different ways to encompass gradients of terragenetic distance.

### Implications for landscape ecology

One of the most important implications of the terragenetic model is that individual fragments are not independent units to use in comparative analyses because they have shared histories. This non-independence of fragment communities may demand a new approach to analysing the distribution of species across fragmented landscapes, just as recognition of the shared history of species demanded a new approach to analysing the distribution of species traits across phylogenies (Felsenstein 1985) and patterns of extinction threat across species (reviewed by Purvis 2008). The similar nature of terragenies and phylogenies ensures that the toolbox of statistical methods developed to quantify and analyse phylogenies and phylogenetically correlated data may now provide an excellent resource for landscape ecologists.

The use of landscape maps is fundamental in landscape ecology, both for modelling biodiversity patterns within fragmented landscapes and for scaling up those models to estimate the total impact of habitat loss and fragmentation on species and communities (Ewers. 2010). Historical maps of habitat cover have already proven invaluable in understanding present-day patterns of biodiversity (Harding. 1998; Kuussaari. 2009; Wearn. 2012), but one implication arising from terragenetic models is that accurate prediction of biodiversity patterns will require time series maps of habitat cover. Ideally, historical data on habitat change through time would always be used to construct a terrageny, but, in most contexts, detailed historical records will not be available. In these cases, an alternative approach may be to infer the form of the terrageny from present-day patterns of habitat distribution combined with spatially explicit models of historical habitat change. This in turn suggests that landscape ecologists will need to more closely integrate their ecological studies with studies on the dynamics of landscapes themselves, which are driven by social and economic, rather than ecological, concerns (Lambin & Meyfroidt 2011).

## Conclusions

As with any model, the usefulness of the terragenetic model will be in both its ability to accurately describe patterns in data, and its ability to fail in informative ways (Hubbell 2001; Rosindell. 2011). The importance of biological processes is revealed by comparing real-world patterns to those of a neutral baseline (Rosindell. 2011), and we anticipate this will be the primary role of the terragenetic model. Obvious examples include quantifying the role of dispersal ability and other species traits in explaining biodiversity patterns in landscapes that have undergone habitat loss and fragmentation. Nonetheless, the terragenetic approach to predicting biodiversity did generate spatial patterns of community composition that are broadly consistent with expected ecological patterns. The model retains predictions of the SAR such that large fragments typically have more species than small fragments, and it predicts distance-decay in the similarity of communities across space, with the pattern emerging naturally from an understanding of the history of a landscape. Predictions of extinction are lower than those arising from applying the SAR to landscape-scale habitat loss which is in line with expectation (He & Hubbell 2011), and although we did not explore it here, the terragenetic model also makes numerical predictions about patterns of community nestedness along gradients of fragment size (Watling & Donnelly 2006). Working with terragenies also provides non-intuitive predictions that do not arise from looking at the geographical distribution of fragments or the distribution of fragment sizes alone, such as locally endemic species occurring in terragenetically distinct fragments rather than in large fragments.

Accounting for landscape history and quantifying it in a terrageny, and by combining a terrageny with a single biological parameter, the *z*-value of the SAR, we were able to derive a diverse set of predictions about biodiversity patterns in fragmented landscapes. In the same way that ignoring evolutionary history limited our ability to understand patterns of extinction risk among species (Purvis 2008), we suggest that ignoring the spatio-temporal patterns of landscape history may limit our ability to understand the biological consequences of habitat loss and fragmentation.
