# Supplementary figures and images for: Using landscape history to predict biodiversity patterns in fragmented landscapes
Source: Ecol Lett. 2013 Aug 11;16(10):1221–33. doi: 10.1111/ele.12160 (PMC4231225; doi:10.1111/ele.12160)

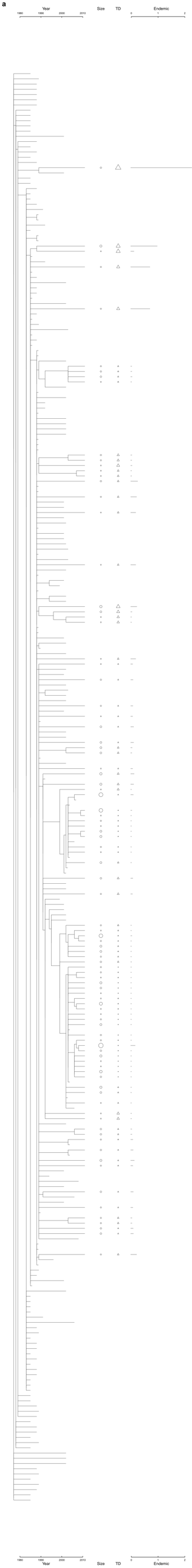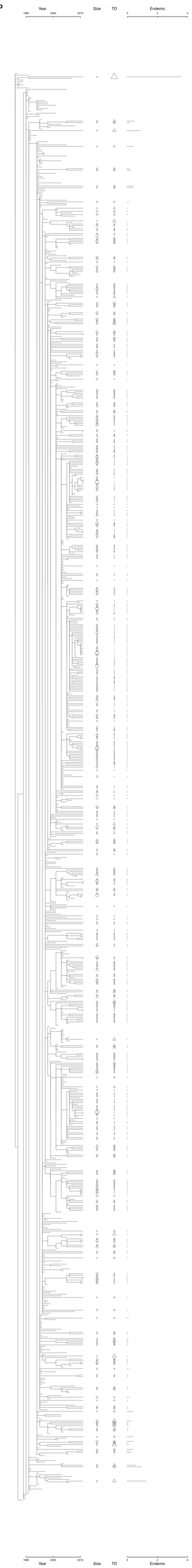

Supplement: Supplementary file 1 [file ele0016-1221-sd1.pdf]
