# Supplementary material for: Using landscape history to predict biodiversity patterns in fragmented landscapes
Source: Ecol Lett. 2013 Aug 11;16(10):1221–33. doi: 10.1111/ele.12160 (PMC4231225; doi:10.1111/ele.12160)
Supplement: Supplementary file 3 [file ele0016-1221-sd3.pdf]

# Using landscape history to predict biodiversity patterns in fragmented landscapes: Online Appendices

Robert M. Ewers, Raphael K. Didham, William D. Pearce,
Veronique Lefebvre, Isabel M.D. Rosa, João Carreiras,
Richard M. Lucas, Daniel C. Reuman

This appendix gives derivations of the expected values and variances presented in the main text. Section A1 precisely describes the assumptions of the terregenesis model. Then we give notation (section A2) and derive distributions for: the number of species in a fragment (section A3); the number of species shared between two fragments (section A4); the total number of species in two fragments (section A5); the similarity ratio of two fragments (section A6); the number of extinct species (section A7); and the number of endemic species (section A8). For convenience, basic laws of probability used in the derivations are summarized in section A9.

## A1 Model assumptions

The terragenesis model assumes:

1. The number of species in the original landscape is fixed;
2. The number of species in any other fragment in the terrageny is a random variable with expected value given by the species area rule (SAR);
3. The $z$ parameter of the SAR model is fixed;
4. Species are mutually independent;
5. The conditional probability of a species being in a fragment, given it is in the parent fragment, is equal to the quotient of the expected number of species in the fragment and the expected number of species in the parent fragment. In particular, the number of species in a fragment given the number of species in the parent fragment is independent of the number of species in all other ancestor fragments (conditional independence).

These assumptions imply that the terrageny tree is constructed as a Bayesian network with binomial random variables.

## A2 Notation

All constants are small letters and random variables are capital letters.

| Notation | Meaning |
|---|---|
| $\mathrm{E}[X]$ | expected value of the random variable X |
| $\mathrm{Var}[X]$ | variance of X |
| $\overline{A}$ | complement of the event $A$ |
| $f_0$ | original landscape |
| $f_{k-1}, f_k, \ldots$ | parent fragment, child fragment |
| $f_{k^{(1)}}, f_{k^{(2)}}, \ldots$ | sibling fragments of generation $k$, all children of $f_{k-1^{(1)}}$ |
| $n_k$ | number of sibling fragments of generation $k$, all children of $f_{k-1^{(1)}}$ |
| $a_k$ | area of $f_k$ |
| $s_0$ | number of species in the original landscape (constant by assumption) |
| $S_k$ | number of species in $f_k$ (random variable), with $k \geq 1$ |
| $F_{k_i}$ | Bernoulli variable describing the event 'species $i$ is present in fragment $f_k$' |
| $I_{k,h}$ | number of species shared by fragments $f_k$ and $f_h$ ($I$ for intersection) |
| $U_{k,h}$ | total number of species present in fragments $f_k$ and $f_h$ ($U$ for union) |
| $SIM_{k,h}$ | similarity of fragments $f_k$ and $f_h$ |
| $X_k$ | number of species from $f_k$ that go extinct by the present day |
| $D_k$ | number of species endemic to $f_k$ |

# A3 Number of species in a fragment

We denote by $F_{k_i}$ the Bernoulli variable for the event 'species $i$ is present in fragment $f_k$', with $i$ denoting a species present in the original landscape, $i = 1, \ldots, s_0$. For all species $i$, we assume that the conditional probability of species $i$ being in fragment $f_k$, given it is in its parent $f_{k-1}$, is equal to the proportion of the expected number of species in $f_k$ to the expected number of species in $f_{k-1}$ (section A1). The probability only depends on the amount of habitat loss in a habitat fragmentation event, and not on species traits. All species are assumed to have the same probability to persist in $f_k$. Thus for all species $i = 1, \ldots, s_0$ and all fragments $f_k$ with $k \geq 1$,

$$\mathrm{P}(F_{k_i} \mid F_{k-1_i}) = \frac{\mathrm{E}[S_k]}{\mathrm{E}[S_{k-1}]}. \tag{1}$$

The expected values, $\mathrm{E}[S_k]$, of the numbers of species in fragments are assumed related to the areas of the fragments through the SAR, $\mathrm{E}[S_k] = c a_k^z$ (section A1), with $z = 0.25$ used here.

From the conditional probability defined in (1) and the chain rule of probability (section A9) we can obtain the probability of a species $i$ being in a fragment $k$. By definition of the terrageny, a species $i$ that is present in $f_k$ is also present in all its ancestor fragments up to the original landscape. Therefore the joint probability of the $F_{k_i}$ events from $f_0$ up to $f_k$ is equal to $F_{k_i}$,

$$\mathrm{P}(F_{k_i}) = \mathrm{P}(F_{k_i} \cap F_{k-1_i} \cap F_{k-2_i} \cap \ldots \cap F_{1_i}), \tag{2}$$

with $f_{k-1}$ denoting the parent fragment of $f_k$. Applying the chain rule of probability,

$$\mathrm{P}(F_{k_i}) = \mathrm{P}(F_{k_i} \mid F_{k-1_i} \cap F_{k-2_i} \cap \ldots \cap F_{1_i}) \times \mathrm{P}(F_{k-1_i} \mid F_{k-2_i} \cap \ldots \cap F_{1_i}) \times \ldots \times \mathrm{P}(F_{1_i}). \tag{3}$$

The probability $\mathrm{P}(F_{k_i} \mid F_{k-1_i})$, that species $i$ is present in the fragment $f_k$ given it is in its parent fragment $f_{k-1}$, is independent of whether species $i$ is present in all anterior ancestors of $f_{k-1}$ (section A1), so

$$\mathrm{P}(F_{k_i} \mid F_{k-1_i} \cap F_{k-2_i} \cap \ldots \cap F_{1_i}) = \mathrm{P}(F_{k_i} \mid F_{k-1_i}). \tag{4}$$

Substituting (4) in (3) for all $k \geq 1$, (3) simplifies to

$$\mathrm{P}(F_{k_i}) = \mathrm{P}(F_{k_i} \mid F_{k-1_i}) \times \mathrm{P}(F_{k-1_i} \mid F_{k-2_i}) \times \ldots \times \mathrm{P}(F_{1_i}) \tag{5}$$

and from (1) we get

$$\begin{aligned} P(F_{k_i}) &= \frac{E[S_k]}{E[S_{k-1}]} \times \frac{E[S_{k-1}]}{E[S_{k-2}]} \times \ldots \times \frac{E[S_1]}{s_0} \\ &= \frac{E[S_k]}{s_0}. \end{aligned} \tag{6}$$

The (unconditional) probability of species $i$ being in fragment $k$ is a Bernoulli random variable. Because species are assumed independent (section A1), the sum

$$S_k = \sum_{i=1}^{s_0} F_{k_i}, \tag{7}$$

equal to the number of species in fragment $k$, is distributed as Binomial$(s_0, p_k)$ for $k \geq 1$, with $p_k = E[S_k]/s_0$, and therefore has variance

$$\begin{aligned} \mathrm{Var}[S_k] &= s_0 p_k \cdot (1 - p_k) \\ &= E[S_k] \cdot \left(1 - \frac{E[S_k]}{s_0}\right) \end{aligned} \tag{8}$$

(section A9). The proportion $S_k/s_0$ has $\mathrm{Var}[\frac{S_k}{so}] = \frac{E[S_k]}{s_0^2} \cdot \left(1 - \frac{E[S_k]}{s_0}\right)$.

# A4 Number of species shared between two fragments

We consider two distinct fragments $f_k$ and $f_h$ anywhere in the terrageny, with $f_r$ their most recent common ancestor. $f_r$ can be $f_0$. The random variable $I_{k,h}$ is defined to be the number of species that $f_k$ and $f_h$ have in common. It is the sum, over the $s_0$ species in $f_0$, of the joint distribution of $F_{k_i}$ and $F_{h_i}$ describing the event 'species $i$ is present in $f_k$ and in $f_h$':

$$I_{k,h} = \sum_{i=1}^{s_0} F_{k_i} \cap F_{h_i}. \tag{9}$$

From the chain rule,

$$P(F_{k_i} \cap F_{h_i}) = P(F_{k_i} \mid F_{h_i}) \times P(F_{h_i}). \tag{10}$$

But $P(F_{k_i} \mid F_{h_i}) = P(F_{k_i} \mid F_{r_i})$ by the assumptions of the model (section A1), and it is straightforward to see that (6) generalizes to

$$P(F_{k_i} \mid F_{g_i}) = \frac{E[S_k]}{E[S_g]} \tag{11}$$

for any ancestor fragment $f_g$ of $f_k$. So

$$\begin{aligned} P(F_{k_i} \cap F_{h_i}) &= P(F_{k_i} \mid F_{r_i}) \times P(F_{h_i}) \\ &= \frac{E[S_k] \cdot E[S_h]}{E[S_r] \cdot s_0}. \end{aligned} \tag{12}$$

From assumptions of independence of species and fragments (section A1) the $F_{k_i} \cap F_{h_i}$ and $F_{k_j} \cap F_{h_j}$ are mutually independent for all $i \neq j$, so the $F_{k_i} \cap F_{h_i}$ are independent identically distributed (i.i.d.) Bernoulli variables of parameter

$$p_{k \cap h} = \frac{E[S_k] \cdot E[S_h]}{E[S_r] \cdot s_0}. \tag{13}$$

Their sum $I_{k,h}$ follows a Binomial distribution of parameters $s_0$ and $p_{k\cap h}$, with expected value

$$\begin{aligned} \mathrm{E}[I_{k,h}] &= s_0 \cdot p_{k\cap h} \\ &= \frac{\mathrm{E}[S_k] \cdot \mathrm{E}[S_h]}{\mathrm{E}[S_r]} \end{aligned} \tag{14}$$

and variance

$$\begin{aligned} \mathrm{Var}[I_{k,h}] &= s_0 \cdot p_{k\cap h} \cdot (1 - p_{k\cap h}) \\ &= \mathrm{E}[I_{k,h}] \cdot \left(1 - \frac{\mathrm{E}[I_{k,h}]}{s_0}\right). \end{aligned} \tag{15}$$

# A5 Total number of species in two fragments

The total number of species in fragments $f_k$ and $f_h$, denoted $U_{k,h}$, corresponds to the size of the union of the two sets of species. Again we start by defining Bernoulli variables for the event 'species $i$ is in $f_k$ or in $f_h$ or both':

$$\begin{aligned} \mathrm{P}(F_{k_i} \cup F_{h_i}) &= \mathrm{P}(F_{k_i}) + \mathrm{P}(F_{h_i}) - \mathrm{P}(F_{k_i} \cap F_{h_i}) \\ &= p_k + p_h - p_{k\cap h}. \end{aligned} \tag{16}$$

The sum of the Bernoulli variables over the $s_0$ species in $f_0$ gives the number of species in the union:

$$U_{k,h} = \sum_{i=1}^{s_0} F_{k_i} \cup F_{h_i}. \tag{17}$$

The $F_{k_i} \cup F_{h_i}$ are i.i.d. Bernoulli variables and their sum $U_{k,h}$ follows a Binomial distribution of parameters $s_0$ and $p_{k\cup h} = p_k + p_h - p_{k\cap h}$. The expected value and variance of $U_{k,h}$ are

$$\begin{aligned} \mathrm{E}[U_{k,h}] &= s_0 \cdot p_{k\cup h} = s_0 \cdot (p_k + p_h - p_{k\cap h}) \\ &= \mathrm{E}[S_k] + \mathrm{E}[S_h] - \frac{\mathrm{E}[S_k] \cdot \mathrm{E}[S_h]}{\mathrm{E}[S_r]} \end{aligned} \tag{18}$$

and

$$\begin{aligned} \mathrm{Var}[U_{k,h}] &= s_0 \cdot p_{k\cup h} \cdot (1 - p_{k\cup h}) \\ &= \mathrm{E}[U_{k,h}] \cdot \left(1 - \frac{\mathrm{E}[U_{k,h}]}{s_0}\right). \end{aligned} \tag{19}$$

# A6 Similarity of two fragments

The Jaccard similarity between two sets is defined as the ratio of the size of their intersection over the size of their union. We denote by $SIM_{k,h}$ the Jaccard similarity between fragments $f_k$ and $f_h$,

$$SIM_{k,h} = \frac{I_{k,h}}{U_{k,h}}. \tag{20}$$

The random variables $I_{k,h}$ and $U_{k,h}$ are supported on the integers $0, \ldots, s_0$, so the quotient is supported on the set of fractions $a/b$ with $a$ and $b$ in $0, \ldots, s_0$ and $a \leq b$. $SIM_{k,h}$ can be undefined if $U_{k,h}$ is zero, an event that has probability $(1 - p_{k\cup h})^{s_0}$, typically very small.

The probability of $SIM_{k,h}$ being equal to a ratio $q$ is the sum of the probabilities of the events $(I_{k,h} = i, U_{k,h} = u)$ over pairs $i$ and $u$ such that $i/u = q$,

$$\mathrm{P}(SIM_{k,h} = q) = \sum_{\substack{(i,u) \text{ such that } \frac{i}{u}=q \\ i\in[0,u], u\in[1,s_0]}} \mathrm{P}(I_{k,h} = i, U_{k,h} = u). \tag{21}$$

The chain rule can be applied:

$$\mathrm{P}(I_{k,h} = i, U_{k,h} = u) = \mathrm{P}(I_{k,h} = i \mid U_{k,h} = u) \times \mathrm{P}(U_{k,h} = u). \tag{22}$$

The second term in the product is known because the distribution of the union size is known (section A5). The first term in the product can be found using similar reasoning to previous sections, as follows. First, by the chain rule,

$$\mathrm{P}(F_{k_i} \cap F_{h_i} \mid F_{k_i} \cup F_{h_i}) = \frac{\mathrm{P}((F_{k_i} \cap F_{h_i}) \cap (F_{k_i} \cup F_{h_i}))}{\mathrm{P}(F_{k_i} \cup F_{h_i})} = \frac{\mathrm{P}(F_{k_i} \cap F_{h_i})}{\mathrm{P}(F_{k_i} \cup F_{h_i})} = \frac{\mathrm{E}[I_{k,h}]/s_0}{\mathrm{E}[U_{k,h}]/s_0}. \tag{23}$$

Therefore, by independence of species,

$$(I_{k,h} \mid U_{k,h} = u) \sim \text{Binomial}(u, p_{I|U}) \tag{24}$$

where

$$p_{I|U} = \frac{\mathrm{E}[I_{k,h}]}{\mathrm{E}[U_{k,h}]}. \tag{25}$$

Therefore the expected value and variance of the number of shared species given the total number of species in $f_k$ and $f_h$ are

$$\begin{aligned} \mathrm{E}[I_{k,h} \mid U_{k,h} = u] &= u \cdot p_{I|U} \\ &= u \cdot \frac{\mathrm{E}[I_{k,h}]}{\mathrm{E}[U_{k,h}]} \end{aligned} \tag{26}$$

$$\begin{aligned} \mathrm{Var}[I_{k,h} \mid U_{k,h} = u] &= u \cdot p_{I|U} \cdot (1 - p_{I|U}) \\ &= u \cdot \frac{\mathrm{E}[I_{k,h}]}{\mathrm{E}[U_{k,h}]} \cdot \left(1 - \frac{\mathrm{E}[I_{k,h}]}{\mathrm{E}[U_{k,h}]}\right). \end{aligned} \tag{27}$$

Because $SIM_{k,h}$ is not defined with probability $(1 - p_{k\cup h})^{s_0}$, it has no expected value in the strictest sense. But because $(1 - p_{k\cup h})^{s_0}$ is typically so small, it makes sense to denote by $\mathrm{E}[SIM_{k,h}]$ the expected value of $SIM_{k,h}$ conditioned on it being defined. We then have, by the definition of the expected value and equation (21),

$$\begin{aligned} \mathrm{E}[SIM_{k,h}] &= \frac{1}{1 - (1 - p_{k\cup h})^{s_0}} \sum_q q \cdot \mathrm{P}(SIM_{k,h} = q) \\ &= \frac{1}{1 - (1 - p_{k\cup h})^{s_0}} \sum_{u=1}^{s_0} \sum_{i=0}^{u} \frac{i}{u} \cdot \mathrm{P}(I_{k,h} = i, U_{k,h} = u). \end{aligned} \tag{28}$$

Replacing with (22), we get

$$\begin{aligned} \mathrm{E}[SIM_{k,h}] &= \frac{1}{1 - (1 - p_{k\cup h})^{s_0}} \sum_{u=1}^{s_0} \sum_{i=0}^{u} \frac{i}{u} \cdot \mathrm{P}(I_{k,h} = i \mid U_{k,h} = u) \cdot \mathrm{P}(U_{k,h} = u) \\ &= \frac{1}{1 - (1 - p_{k\cup h})^{s_0}} \sum_{u=1}^{s_0} \frac{1}{u} \cdot \mathrm{P}(U_{k,h} = u) \cdot \sum_{i=0}^{u} i \cdot \mathrm{P}(I_{k,h} = i \mid U_{k,h} = u) \end{aligned} \tag{29}$$

The sum over $i$ is the expectation of $I_{k,h} \mid U_{k,h} = u$, determined in equation (26). Replacing,

$$\begin{aligned}
\mathrm{E}[SIM_{k,h}] &= \frac{1}{1-(1-p_{k\cup h})^{s_0}} \sum_{u=1}^{s_0} \frac{1}{u} \cdot \mathrm{P}(U_{k,h}=u) \cdot u \cdot p_{I|U} \\
&= \frac{p_{I|U}}{1-(1-p_{k\cup h})^{s_0}} \cdot \sum_{u=1}^{s_0} \mathrm{P}(U_{k,h}=u) \qquad (30)\\
&= p_{I|U}. \qquad (31)
\end{aligned}$$

$\mathrm{P}(U_{k,h}=0) = (1-p_{k\cup h})^{s_0}$ varies with $\mathrm{E}[S_k], \mathrm{E}[S_h], \mathrm{E}[S_r]$ and $s_0$. From simulations, if the sum $\mathrm{E}[S_k] + \mathrm{E}[S_h] > 10$, then $\mathrm{P}(U_{k,h}=0) < 0.001$.

The notation $\mathrm{Var}[SIM_{k,h}]$ here is used to denote variance conditional on $SIM_{k,h}$ being defined. To obtain the variance we use the well known expression

$$\mathrm{Var}[SIM_{k,h}] = \mathrm{E}[SIM_{k,h}^2] - \mathrm{E}[SIM_{k,h}]^2, \qquad (32)$$

and compute the expected square ratio using similar reasoning to equations (28) and (29):

$$\begin{aligned}
\mathrm{E}[SIM_{k,h}^2] &= \frac{1}{1-(1-p_{k\cup h})^{s_0}} \sum_{u=1}^{s_0} \sum_{i=0}^{u} \frac{i^2}{u^2} \cdot \mathrm{P}(I_{k,h}=i, U_{k,h}=u) \\
&= \frac{1}{1-(1-p_{k\cup h})^{s_0}} \sum_{u=1}^{s_0} \frac{1}{u^2} \cdot \mathrm{P}(U_{k,h}=u) \cdot \sum_{i=0}^{u} i^2 \cdot \mathrm{P}(I_{k,h}=i \mid U_{k,h}=u). \qquad (33)
\end{aligned}$$

The sum over $i$ is the expected square intersection size conditioned on $u$. We can obtain its value from the variance of $I_{k,h} \mid U_{k,h} = u$ given in equation (27) and the square of its expected value (26):

$$\begin{aligned}
\mathrm{E}[(I_{k,h} \mid U_{k,h})^2] &= \mathrm{Var}[I_{k,h} \mid U_{k,h}] + \mathrm{E}[I_{k,h} \mid U_{k,h}]^2 \\
&= u \cdot p_{I|U} \cdot (1-p_{I|U}) + u^2 \cdot p_{I|U}^2. \qquad (34)
\end{aligned}$$

Replacing in (33), distributing and noting the expression of the inverse union expectation we obtain:

$$\begin{aligned}
\mathrm{E}[SIM_{k,h}^2] &= \frac{1}{1-(1-p_{k\cup h})^{s_0}} \sum_{u=1}^{s_0} \frac{1}{u^2} \cdot \mathrm{P}(U_{k,h}=u) \cdot \left[u \cdot p_{I|U} \cdot (1-p_{I|U}) + u^2 \cdot p_{I|U}^2\right] \\
&= \frac{1}{1-(1-p_{k\cup h})^{s_0}} \left[\sum_{u=1}^{s_0} \frac{1}{u^2} \cdot \mathrm{P}(U_{k,h}=u) \cdot u \cdot p_{I|U} \cdot (1-p_{I|U}) \right.\\
&\quad \left. + \sum_{u=1}^{s_0} \frac{1}{u^2} \cdot \mathrm{P}(U_{k,h}=u) \cdot u^2 \cdot p_{I|U}^2 \right] \\
&= \frac{1}{1-(1-p_{k\cup h})^{s_0}} \left[p_{I|U} \cdot (1-p_{I|U}) \sum_{u=1}^{s_0} \frac{1}{u} \mathrm{P}(U_{k,h}=u) + p_{I|U}^2 \cdot \sum_{u=1}^{s_0} \mathrm{P}(U_{k,h}=u)\right] \\
&= p_{I|U} \cdot (1-p_{I|U}) \cdot \mathrm{E}\left[\frac{1}{U_{k,h}}\right] + p_{I|U}^2, \qquad (35)
\end{aligned}$$

where $\mathrm{E}[1/U_{k,h}]$ denotes the expectation of $1/U_{k,h}$ conditional on it being defined. Replacing in the definition of the variance, equation (32), we get

$$\begin{aligned}
\mathrm{Var}[SIM_{k,h}] &= p_{I|U} \cdot (1-p_{I|U}) \cdot \mathrm{E}\left[\frac{1}{U_{k,h}}\right] + p_{I|U}^2 - p_{I|U}^2 \qquad (36)\\
&= p_{I|U} \cdot (1-p_{I|U}) \cdot \mathrm{E}\left[\frac{1}{U_{k,h}}\right]. \qquad (37)
\end{aligned}$$

$\mathrm{E}\left[\frac{1}{U_{k,h}}\right]$ also needs to be approximated as there is no closed form for this expectation. There is ongoing research to find expansions of the inverse moments of binomial variables [Marciniak and Wesolowski, 1999, Audenaert, 2008, Znidaric, 2009]. If $X \sim \mathrm{Binomial}(n, p)$ and $\mathrm{E}[X]$ is large then the first inverse moment can easily be approximated as

$$\lim_{\mathrm{E}[X]\to+\infty} E\left[\frac{1}{X}\right] = \frac{1}{\mathrm{E}[X]}. \tag{38}$$

This approximation is poor, however, for small $\mathrm{E}[X]$:

$$\lim_{\mathrm{E}[X]\to 0} \mathrm{E}\left[\frac{1}{X}\right] = 0 \neq \lim_{\mathrm{E}[X]\to 0} \frac{1}{\mathrm{E}[X]} = +\infty. \tag{39}$$

From computations over a range of $n$ ($< 10^5$) and $p$ ($> 10^{-5}$) values the following bounds can be given:

$$\begin{aligned}
\mathrm{E}[X] > 30 \;&\Rightarrow\; \left|\mathrm{E}\left[\frac{1}{X}\right] - \frac{1}{\mathrm{E}[X]}\right| \lesssim 0.001 \\
\mathrm{E}[X] > 10 \;&\Rightarrow\; \left|\mathrm{E}\left[\frac{1}{X}\right] - \frac{1}{\mathrm{E}[X]}\right| \lesssim 0.01 \\
\mathrm{E}[X] > 2 \;&\Rightarrow\; \left|\mathrm{E}\left[\frac{1}{X}\right] - \frac{1}{\mathrm{E}[X]}\right| \lesssim 0.1.
\end{aligned}$$

For small values of $n$ the difference between $\mathrm{E}\left[\frac{1}{X}\right]$ and $\frac{1}{\mathrm{E}[X]}$ can also be very small if $p$ is large enough. In the case where $X = U_{k,h}$, the limit (38) will typically be good enough, at least for patches of non-negligible size and species-rich taxonomic groups.

If the approximation from equation (38) is not sufficient, i.e., in cases of small numbers of species because of drastic habitat loss, $\mathrm{E}\left[\frac{1}{U_{k,h}}\right]$ can be computed from the exact formula

$$(1-(1-p_{k\cup h})^{s_0}) \cdot \mathrm{E}\left[\frac{1}{U_{k,h}}\right] = \sum_{u=1}^{s_0} \frac{1}{u}\binom{s_0}{u} \cdot p_{k\cup h}^{u} \cdot (1-p_{k\cup h})^{s_0-u}. \tag{40}$$

The following sum provides a close fit with a shorter computation time [Marciniak and Wesolowski, 1999]:

$$(1-(1-p_{k\cup h})^{s_0}) \cdot \mathrm{E}\left[\frac{1}{U_{k,h}}\right] = \sum_{u=1}^{s_0} \frac{(1-p_{k\cup h})^{s_0-u}}{u} - (1-p_{k\cup h})^{s_0} \sum_{u=1}^{s_0} \frac{1}{u}. \tag{41}$$

For even faster computation but slightly larger error [Znidaric, 2009]:

$$(1-q_{k\cup h}^{s_0}) \cdot \mathrm{E}\left[\frac{1}{U_{k,h}}\right] = \frac{1}{\mathrm{E}[U_{k,h}]} \cdot \left(1 + \frac{q_{k\cup h}}{\mathrm{E}[U_{k,h}]} + \frac{q_{k\cup h}(1+q_{k\cup h})}{\mathrm{E}[U_{k,h}]^2} + \frac{q_{k\cup h}(1+4q_{k\cup h}+q_{k\cup h}^2)}{\mathrm{E}[U_{k,h}]^3} + \dots\right), \tag{42}$$

with $q_{k\cup h} = 1 - p_{k\cup h}$.

Given the simplest approximation, from (38), the variance of the Jaccard similarity ratio can then be expressed as:

$$\begin{aligned}
\mathrm{Var}[SIM_{k,h}] &\approx p_{I|U} \cdot (1-p_{I|U}) \cdot \frac{1}{\mathrm{E}[U_{k,h}]} \\
&= \frac{\mathrm{E}[I_{k,h}]}{\mathrm{E}[U_{k,h}]^2}\left(1 - \frac{\mathrm{E}[I_{k,h}]}{\mathrm{E}[U_{k,h}]}\right) \qquad (43) \\
&= \frac{\mathrm{E}[SIM_{k,h}](1-\mathrm{E}[SIM_{k,h}])}{\mathrm{E}[U_{k,h}]}. \qquad (44)
\end{aligned}$$

## A7 Number of extinct species

A species $i$ present in $f_k$ goes extinct from the lineage of $f_k$ if, for each child fragment $f_{k+1^{(c)}}$, the species is either not present in $f_{k+1^{(c)}}$, or is present, but goes extinct subsequently from the lineage of $f_{k+1^{(c)}}$. We denote by $X_{k_i}$ the Bernoulli variable describing the event 'species i is present in $f_k$ but goes extinct from the lineage of $f_k$'. $X_{k_i}$ is defined for all species in the landscape, $i = 1, \dots, s_0$. A species that goes extinct from the lineage of a fragment is present in that fragment by definition, therefore the probability of $X_{k_i}$ can be expressed as

$$\begin{aligned} \mathrm{P}(X_{k_i}) &= \mathrm{P}(X_{k_i} \cap F_{k_i}) \\ &= \mathrm{P}(X_{k_i} | F_{k_i}) \times \mathrm{P}(F_{k_i}). \end{aligned} \tag{45}$$

If $c = 1, \dots, n_{k+1}$ index the children $f_{k+1^{(c)}}$ of $f_k$, then $X_{k_i}|F_{k_i}$ is expressed in a recursive manner using $F_{k+1_i^{(c)}}$ and $X_{k+1_i^{(c)}}$ conditioned on $F_{k_i}$:

$$X_{k_i}|F_{k_i} = \bigcap_{c=1}^{n_{k+1}} ((\overline{F_{k+1_i^{(c)}}} \mid F_{k_i}) \cup (F_{k+1_i^{(c)}} \cap X_{k+1_i^{(c)}} \mid F_{k_i})). \tag{46}$$

By independence assumptions of the model (section A1),

$$\mathrm{P}(X_{k_i}|F_{k_i}) = \prod_{c=1}^{n_{k+1}} \mathrm{P}((\overline{F_{k+1_i^{(c)}}} \mid F_{k_i}) \cup (F_{k+1_i^{(c)}} \cap X_{k+1_i^{(c)}} \mid F_{k_i})) \tag{47}$$

The events 'species $i$ is not in $f_{k+1^{(c)}}$' and 'species $i$ is in $f_{k+1^{(c)}}$, but goes extinct subsequently from its lineage' are disjoint, so the probability of their union is the sum of their probabilities:

$$\mathrm{P}(X_{k_i}|F_{k_i}) = \prod_{c=1}^{n_{k+1}} \left[\mathrm{P}(\overline{F_{k+1_i^{(c)}}} \mid F_{k_i}) + \mathrm{P}(F_{k+1_i^{(c)}} \cap X_{k+1_i^{(c)}} \mid F_{k_i})\right]. \tag{48}$$

From (1),

$$\begin{aligned} \mathrm{P}(\overline{F_{k+1_i^{(c)}}} \mid F_{k_i}) &= 1 - \mathrm{P}(F_{k+1_i^{(c)}} \mid F_{k_i}) \\ &= 1 - \frac{\mathrm{E}[S_{k+1^{(c)}}]}{\mathrm{E}[S_k]}. \end{aligned} \tag{49}$$

From the chain rule (section A9) and the conditional independence of presence in ancestors given the presence in the parent (section A1), the probability that species $i$ is present in $f_{k+1^{(c)}}$ and goes extinct from its lineage given it is in $f_k$ is

$$\begin{aligned} \mathrm{P}(F_{k+1_i^{(c)}} \cap X_{k+1_i^{(c)}} \mid F_{k_i}) &= \mathrm{P}(X_{k+1_i^{(c)}} \mid F_{k+1_i^{(c)}} \cap F_{k_i}) \times \mathrm{P}(F_{k+1_i^{(c)}} \mid F_{k_i}) \\ &= \mathrm{P}(X_{k+1_i^{(c)}} \mid F_{k+1_i^{(c)}}) \times \mathrm{P}(F_{k+1_i^{(c)}} \mid F_{k_i}) && (50) \\ &= \frac{\mathrm{E}[X_{k+1^{(c)}}]}{\mathrm{E}[S_{k+1^{(c)}}]} \times \frac{\mathrm{E}[S_{k+1^{(c)}}]}{\mathrm{E}[S_k]} \\ &= \frac{\mathrm{E}[X_{k+1^{(c)}}]}{\mathrm{E}[S_k]} && (51) \end{aligned}$$

The probability of species $i$ going extinct from the lineage of $f_{k+1^{(c)}}$ given it is in $f_{k+1^{(c)}}$ is the expected number of species that are in $f_{k+1^{(c)}}$ but go extinct from the lineage of $f_{k+1^{(c)}}$, divided by the expected number of species in $f_{k+1^{(c)}}$, because all species have an equal probability of going extinct from the lineage of $f_{k+1^{(c)}}$. We can therefore recursively express the probability

that species $i$ in $f_k$ goes extinct from the lineage of $f_k$ using the expected number of extinctions from the child fragments of $f_k$:

$$\begin{aligned}
\mathrm{P}(X_{k_i}|F_{k_i}) &= \prod_{c=1}^{n_{k+1}} \left[1 - \frac{\mathrm{E}[S_{k+1^{(c)}}]}{\mathrm{E}[S_k]} + \mathrm{P}(X_{k+1_i^{(c)}} \mid F_{k+1_i^{(c)}}) \times \frac{\mathrm{E}[S_{k+1^{(c)}}]}{\mathrm{E}[S_k]}\right] \quad (52)\\
&= \prod_{c=1}^{n_{k+1}} \left[1 - \frac{\mathrm{E}[S_{k+1^{(c)}}]}{\mathrm{E}[S_k]} + \frac{\mathrm{E}[X_{k+1^{(c)}}]}{\mathrm{E}[S_k]}\right]\\
&= \frac{1}{\mathrm{E}[S_k]^{n_{k+1}}} \prod_{c=1}^{n_{k+1}} \left[\mathrm{E}[S_k] - \mathrm{E}[S_{k+1^{(c)}}] + \mathrm{E}[X_{k+1^{(c)}}]\right]. \quad (53)
\end{aligned}$$

The equation for $e_k$ in the main text follows from (52).

We denote $p_{X_k|F_k} = \mathrm{P}(X_{k_i}|F_{k_i})$ so that

$$\mathrm{P}(X_{k_i}) = p_{X_k|F_k} \cdot \mathrm{P}(F_{k_i}), \quad (54)$$

and from (6) we get

$$\mathrm{P}(X_{k_i}) = p_{X_k|F_k} \cdot \frac{\mathrm{E}[S_k]}{s_0}. \quad (55)$$

$X_{k_i}$ is a Bernoulli variable of probability $p_{X_k|F_k} \cdot \frac{\mathrm{E}[S_k]}{s_0}$. The number $X_k$ of species that are in $f_k$ but that go extinct from the lineage of $f_k$ is the sum of the i.i.d. Bernoulli variables $X_{k_i}$ over the number $s_0$ of species present in the original landscape:

$$X_k = \sum_{i=1}^{s_0} X_{k_i}. \quad (56)$$

$X_k$ follows a Binomial distribution of parameters $s_0$ and $p_{X_k|F_k} \cdot \frac{\mathrm{E}[S_k]}{s_0}$. We obtain the expected value and variance of $X_k$ from the properties of the Binomial distribution:

$$\begin{aligned}
\mathrm{E}[X_k] &= s_0 \cdot p_{X_k|F_k} \cdot \frac{\mathrm{E}[S_k]}{s_0}\\
&= p_{X_k|F_k} \cdot \mathrm{E}[S_k]\\
&= \frac{1}{\mathrm{E}[S_k]^{n_{k+1}-1}} \prod_{c=1}^{n_{k+1}} [\mathrm{E}[S_k] - \mathrm{E}[S_{k+1^{(c)}}] + \mathrm{E}[X_{k+1^{(c)}}]] \quad (57)
\end{aligned}$$

$$\begin{aligned}
\mathrm{Var}[X_k] &= s_0 \cdot p_{X_k|F_k} \cdot \frac{\mathrm{E}[S_k]}{s_0} \cdot \left(1 - p_{X_k|F_k} \cdot \frac{\mathrm{E}[S_k]}{s_0}\right)\\
&= \mathrm{E}[X_k] \cdot \left(1 - \frac{\mathrm{E}[X_k]}{s_0}\right). \quad (58)
\end{aligned}$$

The expected value and variance of $X_k$ are computed recursively. The number of extinctions for present time fragments that have not been split is 0; this is the recursive base case. The expected number of extinctions that occurred in the whole landscape is obtained by setting $k$ to 0. The variance of the proportion of species from the original landscape that go extinct by the present day, a quantity given in the main text, is

$$\begin{aligned}
\frac{\mathrm{Var}[X_0]}{s_0^2} &= \frac{\mathrm{E}[X_0]}{s_0^2}(1 - \frac{\mathrm{E}[X_0]}{s_0})\\
&= \frac{e_0}{s_0}(1 - e_0),
\end{aligned}$$

where $e_0$ is defined in the main text.

# A8 Number of endemic species

A species $i$ is defined to be "dominated" by a fragment $f_k$ in the terrageny if $i$ is in $f_k$ and the only present-day patches containing $i$ (if any) are direct descendants of $f_k$. If $f_k$ is a present-day patch, then this is the same as saying $i$ is endemic to $f_k$ within the modern landscape. All $s_0$ species present in the original landscape are dominated by $f_0$. And reciprocally a species dominated by a fragment $f_k$ is also dominated by its parent $f_{k-1}$ and by all its ancestors up to the original landscape. $D_{k_i}$ denotes the event 'species $i$ is dominated by fragment $f_k$'. From the chain rule of probability and the conditional independence of a fragment to all its ancestors given its parent (section A1),

$$\begin{aligned} \mathrm{P}(D_{k_i}) &= \mathrm{P}(D_{k_i} \cap D_{k-1_i} \cap D_{k-2_i} \cap \ldots \cap D_{1_i}) & (59)\\ &= \mathrm{P}(D_{k_i} \mid D_{k-1_i}) \times \mathrm{P}(D_{k-1_i} \mid D_{k-2_i}) \times \ldots \times \mathrm{P}(D_{1_i}). & (60)\end{aligned}$$

Thus we can obtain the probability for species $i$ to be dominated by $f_k$ by evaluating for $f_k$ and each other patch in the ancestry of $f_k$ the conditional probability that $i$ is dominated by the patch given that it is dominated by its parent.

A species $i$ is dominated by child fragment $f_{k^{(1)}}$ given that it is dominated by $f_{k-1^{(1)}}$ (i.e., $D_{k_i^{(1)}} \mid D_{k-1_i^{(1)}}$) if it is present in $f_{k^{(1)}}$ and if, for any $c = 2, \ldots, n_k$, it is not present in $f_{k^{(c)}}$ or is but then goes extinct from the lineage of $f_{k^{(c)}}$. Recall that here $c$ indexes the sibling fragments of $f_{k^{(1)}}$, children of $f_{k-1^{(1)}}$. This conditional event can be expressed with extinction and presence events, respectively $X_{k_i^{(c)}}$ and $F_{k_i^{(c)}}$, for the $n_k$ children of $f_{k-1^{(1)}}$:

$$(D_{k_i^{(1)}} \mid D_{k-1_i^{(1)}}) = (F_{k_i^{(1)}} \mid D_{k-1_i^{(1)}}) \bigcap_{c=2}^{n_k} ((\overline{F_{k_i^{(c)}}} \mid D_{k-1_i^{(1)}}) \cup (F_{k_i^{(c)}} \cap X_{k_i^{(c)}} \mid D_{k-1_i^{(1)}})). \quad (61)$$

From mutual independence of fragment-specific events and by disjointness of events, the probability of $(D_{k_i^{(1)}} \mid D_{k-1_i^{(1)}})$ is

$$\mathrm{P}(D_{k_i^{(1)}} \mid D_{k-1_i^{(1)}}) = \mathrm{P}(F_{k_i^{(1)}} \mid D_{k-1_i^{(1)}}) \prod_{c=2}^{n_k} (\mathrm{P}(\overline{F_{k_i^{(c)}}} \mid D_{k-1_i^{(1)}}) + \mathrm{P}(F_{k_i^{(c)}} \cap X_{k_i^{(c)}} \mid D_{k-1_i^{(1)}})). \quad (62)$$

The probability that species $i$ is in $f_{k^{(1)}}$ given it is dominated by its parent, $f_{k-1^{(1)}}$, is the same as the probability $i$ is present in $f_{k^{(1)}}$ given it is present in $f_{k-1^{(1)}}$. So

$$\begin{aligned} \mathrm{P}(F_{k_i^{(1)}} \mid D_{k-1_i^{(1)}}) &= \mathrm{P}(F_{k_i^{(1)}} \mid F_{k-1_i^{(1)}}) \\ &= \frac{\mathrm{E}[S_{k^{(1)}}]}{\mathrm{E}[S_{k-1^{(1)}}]}. & (63)\end{aligned}$$

Applying this reasoning to all events in (62) conditioned on $D_{k-1_i^{(1)}}$ implies that

$$\begin{aligned} \mathrm{P}(D_{k_i^{(1)}} \mid D_{k-1_i^{(1)}}) &= \mathrm{P}(F_{k_i^{(1)}} \mid F_{k-1_i^{(1)}}) \prod_{c=2}^{n_k} \left[\mathrm{P}(\overline{F_{k_i^{(c)}}} \mid F_{k-1_i^{(1)}}) + \mathrm{P}(F_{k_i^{(c)}} \cap X_{k_i^{(c)}} \mid F_{k-1_i^{(1)}})\right] \\ &= \mathrm{P}(F_{k_i^{(1)}} \mid F_{k-1_i^{(1)}}) \prod_{c=2}^{n_k} \Big[1 - \mathrm{P}(F_{k_i^{(c)}} \mid F_{k-1_i^{(1)}}) + \\ &\qquad \mathrm{P}(X_{k_i^{(c)}} \mid F_{k_i^{(c)}}) \times \mathrm{P}(F_{k_i^{(c)}} \mid F_{k-1_i^{(1)}})\Big] \\ &= \frac{\mathrm{E}[S_{k^{(1)}}]}{\mathrm{E}[S_{k-1^{(1)}}]} \prod_{c=2}^{n_k} \left[1 - \frac{\mathrm{E}[S_{k^{(c)}}]}{\mathrm{E}[S_{k-1^{(1)}}]} + \frac{\mathrm{E}[X_{k^{(c)}}]}{\mathrm{E}[S_{k-1^{(1)}}]}\right] & (64)\end{aligned}$$

Again with a change of index the terms of the product are the same as for the extinction event in equation (53). However the product is only over the siblings of $f_{k^{(1)}}$ and does not include $f_{k^{(1)}}$ itself, whereas in the case of extinctions the product was over all children.

Using the conditional probabilities $\mathrm{P}(D_{k_i^{(1)}} \mid D_{k-1_i^{(1)}})$ and the chain equation (60) we obtain the probability for species $i$ in $f_0$ to be dominated by fragment $f_{k^{(1)}}$:

$$\begin{aligned}
\mathrm{P}(D_{k_i^{(1)}}) &= \prod_{t=1}^{k} \mathrm{P}(D_{t_i^{(1)}} \mid D_{t-1_i^{(1)}}) \\
&= \prod_{t=1}^{k} \left[ \frac{\mathrm{E}[S_{t^{(1)}}]}{\mathrm{E}[S_{t-1^{(1)}}]} \prod_{c=2}^{n_t} \left[ 1 - \frac{\mathrm{E}[S_{t^{(c)}}]}{\mathrm{E}[S_{t-1^{(1)}}]} + \frac{\mathrm{E}[X_{t^{(c)}}]}{\mathrm{E}[S_{t-1^{(1)}}]} \right] \right] \\
&= \frac{\mathrm{E}[S_{k^{(1)}}]}{s_0} \prod_{t=1}^{k} \left[ \prod_{c=2}^{n_t} \left[ 1 - \frac{\mathrm{E}[S_{t^{(c)}}]}{\mathrm{E}[S_{t-1^{(1)}}]} + \frac{\mathrm{E}[X_{t^{(c)}}]}{\mathrm{E}[S_{t-1^{(1)}}]} \right] \right] \\
&= \frac{\mathrm{E}[S_{k^{(1)}}]}{s_0} \prod_{t=1}^{k} \left[ \prod_{c=2}^{n_t} \left[ \frac{1}{\mathrm{E}[S_{t-1^{(1)}}]} \left( \mathrm{E}[S_{t-1^{(1)}}] - \mathrm{E}[S_{t^{(c)}}] + \mathrm{E}[X_{t^{(c)}}] \right) \right] \right] \\
&= \frac{\mathrm{E}[S_{k^{(1)}}]}{s_0} \prod_{t=1}^{k} \left[ \frac{1}{\mathrm{E}[S_{t-1^{(1)}}]^{n_t-1}} \prod_{c=2}^{n_t} \left( \mathrm{E}[S_{t-1^{(1)}}] - \mathrm{E}[S_{t^{(c)}}] + \mathrm{E}[X_{t^{(c)}}] \right) \right]. \qquad (65)
\end{aligned}$$

All species have an equal probability to be dominated by $f_{k^{(1)}}$. For all species $i$ in the original landscape ($i = 1, \ldots, s_0$) the $D_{k_i^{(1)}}$ variables describing the event to be dominated by $f_{k^{(1)}}$ are i.i.d. Bernoulli variables of parameter $p_{D_{k^{(1)}}} = \mathrm{P}(D_{k_i^{(1)}})$. The number $D_{k^{(1)}}$ of species dominated by $f_{k^{(1)}}$ follows a Binomial distribution of parameters $s_0$ and $p_{D_{k^{(1)}}}$. Its expectation and variance are given by:

$$\begin{aligned}
\mathrm{E}[D_{k^{(1)}}] &= s_0 \cdot p_{D_{k^{(1)}}} \\
&= \mathrm{E}[S_{k^{(1)}}] \prod_{t=1}^{k} \left[ \frac{1}{\mathrm{E}[S_{t-1^{(1)}}]^{n_t-1}} \prod_{c=2}^{n_t} \left( \mathrm{E}[S_{t-1^{(1)}}] - \mathrm{E}[S_{t^{(c)}}] + \mathrm{E}[X_{t^{(c)}}] \right) \right] \qquad (66) \\
\mathrm{Var}[D_{k^{(1)}}] &= s_0 \cdot p_{D_{k^{(1)}}} \cdot (1 - p_{D_{k^{(1)}}}) \\
&= \mathrm{E}[D_{k^{(1)}}] \cdot \left( 1 - \frac{\mathrm{E}[D_{k^{(1)}}]}{s_0} \right) \qquad (67)
\end{aligned}$$

# A9 Some basic definitions and laws of probability

$X$ and $Y$ are random variables and $A$, $B$ and $C$ are events. For further information on these laws, see, e.g., Siegrist [1997–2012].

| Topic | Definitions and laws |
|---|---|
| Bernoulli distribution | $Y \sim \mathrm{Bernoulli}(p) \Rightarrow \mathrm{P}(Y=1) = p$ and $\mathrm{P}(Y=0) = 1-p$<br>$\mathrm{E}[Y] = p$<br>$\mathrm{Var}[Y] = p(1-p)$ |
| Binomial distribution | If $Y_1, \ldots, Y_n$ are independent, identically distributed (i.i.d.) Bernoulli variables with success probability p, then<br>$Z = \sum_{k=1}^{n} Y_k \sim \mathrm{Binomial}(n, p)$<br>$\mathrm{E}[Z] = n \cdot p$ |

| | |
|---|---|
| | $\mathrm{Var}[Z] = n \cdot p \cdot (1-p)$ |
| Mean Bernoulli | $n\bar{Y} \sim \mathrm{Binomial}(n, p)$<br>$\mathrm{E}[\bar{Y}] = p$<br>$\mathrm{Var}[\bar{Y}] = \frac{p \cdot (1-p)}{n}$ |
| Expected value<br>Linearity | $\mathrm{E}[Y] = \sum_k k \cdot \mathrm{P}(Y = k)$ for discretely valued $Y$<br>$\mathrm{E}[aX + bY + c] = a\mathrm{E}[X] + b\mathrm{E}[Y] + c$ |
| Variance<br>Of linear combinations | $\mathrm{Var}[Y] = \sum_k \mathrm{P}(Y = k) \cdot (k - \mathrm{E}[Y])^2$<br>$\mathrm{Var}[aX + bY] = a^2\mathrm{Var}[X] + b^2\mathrm{Var}[Y] + 2ab \cdot \mathrm{Cov}[X, Y]$ |
| Chain rule | $\mathrm{P}(A \cap B) = \mathrm{P}(A \mid B) \times \mathrm{P}(B)$<br>$\mathrm{P}(\bigcap_{k=1}^{n} A_k) = \prod_{k=1}^{n} \mathrm{P}(A_k \mid \bigcap_{j=1}^{k-1} A_j)$ |
| Conditional independence | A and B are conditionally independent given C if and only if<br>$\mathrm{P}(A \cap B \mid C) = \mathrm{P}(A \mid C) \times \mathrm{P}(B \mid C)$<br>$\Leftrightarrow \mathrm{P}(A \mid B \cap C) = \mathrm{P}(A \mid C)$ |
| Independence | $\mathrm{P}(A \cap B) = \mathrm{P}(A) \times \mathrm{P}(B) \Leftrightarrow \mathrm{P}(A \mid B) = \mathrm{P}(A)$ |
| Mutual independence | $\mathrm{P}\left(\bigcap_{k=1}^{n} A_k\right) = \prod_{k=1}^{n} \mathrm{P}(A_k)$ |
| Inclusion-exclusion principle | $\mathrm{P}(A \cup B) = \mathrm{P}(A) + \mathrm{P}(B) - \mathrm{P}(A \cap B)$ |
| Disjoint events | $\mathrm{P}(A \cup B) = \mathrm{P}(A) + \mathrm{P}(B)$ |

# References


K. Audenaert. Inverse moments of univariate discrete distributions via the poisson expansion, 2008. `http://arxiv.org/pdf/0809.4155v1.pdf`.

C.M. Marciniak and J. Wesolowski. Asymptotic eulerian expansions for binomial and negative binomial reciprocals. *Proceeding of the American Mathematical Society*, 127:3329–3338, 1999.

K. Siegrist. Virtual laboratories in probability and statistics, 1997–2012. `http://www.math.uah.edu/stat/`.

M. Znidaric. Asymptotic expansion for inverse moments of binomial and poisson distributions. *Open Statistics and Probability Journal*, 1:7–10, 2009.
